# Uncovering the information immunology journals transmitted for COVID-19: A bibliometric and visualization analysis

**DOI:** 10.3389/fimmu.2022.1035151

**Published:** 2022-10-31

**Authors:** Jiefeng Zhao, Jinfeng Zhu, Chao Huang, Xiaojian Zhu, Zhengming Zhu, Qinrong Wu, Rongfa Yuan

**Affiliations:** ^1^Department of General Surgery, The Second Affiliated Hospital of Nanchang University, Nanchang, Jiangxi, China; ^2^Center for Digestive Disease, The Seventh Affiliated Hospital of Sun Yat-sen University, Shenzhen, China; ^3^Department of General Surgery, Yingtan City People’s Hospital, Yingtan, Jiangxi, China

**Keywords:** immunology journal, B-ibliometric analysis, CiteSpace, R-bibliometrix, the coronavirus disease 2019 (COVID-19)

## Abstract

**Background:**

Since the global epidemic of the coronavirus disease 2019 (COVID-19), a large number of immunological studies related to COVID-19 have been published in various immunology journals. However, the results from these studies were discrete, and no study summarized the important immunological information about COVID-19 released by these immunology journals. This study aimed to comprehensively summarize the knowledge structure and research hotspots of COVID-19 published in major immunology journals through bibliometrics.

**Methods:**

Publications on COVID-19 in major immunology journals were obtained from the Web of Science Core Collection. CiteSpace, VOSviewer, and R-bibliometrix were comprehensively used for bibliometric and visual analysis.

**Results:**

1,331 and 5,000 publications of 10 journals with high impact factors and 10 journals with the most papers were included, respectively. The USA, China, England, and Italy made the most significant contributions to these papers. University College London, National Institute of Allergy and Infectious Diseases, Harvard Medical School, University California San Diego, and University of Pennsylvania played a central role in international cooperation in the immunology research field of COVID-19. Yuen Kwok Yung was the most important author in terms of the number of publications and citations, and the H-index. *CLINICAL INFECTIOUS DISEASES* and *FRONTIERS IN IMMUNOLOGY* were the most essential immunology journals. These immunology journals mostly focused on the following topics: “Delta/Omicron variants”, “cytokine storm”, “neutralization/neutralizing antibody”, “T cell”, “BNT162b2”, “mRNA vaccine”, “vaccine effectiveness/safety”, and “long COVID”.

**Conclusion:**

This study systematically uncovered a holistic picture of the current research on COVID-19 published in major immunology journals from the perspective of bibliometrics, which will provide a reference for future research in this field.

## Introduction

As of June 29, 2022, COVID-19 has caused 543,352,927 confirmed cases and 6,331,059 deaths globally (1). Currently, the disease continues to spread rapidly and the world is still struggling with COVID-19, a serious threat to global public health. Since the global pandemic of COVID-19, as an important basic science, immunology has been playing an extremely important role in the pathogenesis, prevention, and treatment of COVID-19: Study has shown that soluble circulating IgG immune complexes are detected in about 80% of patients with severe and critical COVID-19 (2), which can significantly lead to long-term disease progression of COVID-19 (3). A recent study revealed that antibody-dependent enhancement antibodies are produced by SARS-CoV-2 infection and the process can be mediated by at least two different host factors, Fcγ receptor and complement component C1q (4). Activation of the complement system has been identified as part of the COVID-19 clinical syndrome and plays a key role in the pathogenesis and disease severity of COVID-19 (5, 6), and in moderate COVID-19 patients, the expression of classical complement system genes is increased, while in severe patients, the activation of selective complement system genes is increased (7). Complement inhibition against COVID-19 can improve symptoms and reduce mortality by reducing inflammation and preventing lung injury (8–13), especially for C3, C5, and C5a (5, 14, 15). The replication cycle of SARS-CoV-2 also interacts with the autophagy-lysosome pathway in host cells, which is expected to become a new strategy against COVID-19 (16). SARS-CoV-2 infects human cells with the trimeric spike protein by binding to a receptor named angiotensin-converting enzyme II (ACE2) (17, 18), and new ACE2 decoy receptor has been proved to demonstrate remarkable neutralization capacities against diverse prevalent SARS-CoV-2 mutants (19). Studies have also shown that COVID-19 has many similarities with autoimmune diseases such as systemic lupus erythematosus (SLE) (20). Moreover, although accurate information regarding the pros and cons of vaccines is not currently available, various COVID-19 vaccines have shown good efficacy in preventing SARS-CoV-2 infection and reducing disease severity and mortality (21–23). The anti-SARS-CoV-2 monoclonal antibodies demonstrated positive effects in pre-exposure prophylaxis against susceptible variants (24), prevention of progression to severe COVID-19 (25), and reduction of patient mortality (26). In addition, because it can recognize the receptor binding domain (RBD) or other SARS-CoV-2 receptors, nanobodies have also been developed to detect the spike proteins of SARS-CoV-2 variants including Omicron (27), and show the potential of neutralizing SARS-CoV-2 variants (28–31).

The journals of immunology are important platforms for the publication of immunology research, dedicated to publishing all aspects of inflammation and innate immunity, immune receptors, apoptosis, and signaling pathways, antigen presentation, gene regulation and recombination, cellular and systemic immunity, vaccine, immune tolerance, autoimmunity, and tumor immunology. A large number of immunologic research results related to COVID-19 have been published in various immunology journals. However, these research results are discrete and there are no studies to summarize the key immunological information about COVID-19 transmitted by these immunology journals.

Thanks to the fact that bibliometrics can intuitively display the latest progress in a certain research field, sort out research hotspots, and predict the development trend of this field, it has become a very important methodology for researchers to effectively identify and visualize the research development trend. Some researchers have applied bibliometrics to explore the relevant research fields of COVID-19 (32–35). In this study, we tried to conduct a comprehensive and detailed bibliometric analysis of the publications on COVID-19 published in major immunologic journals, and determine the current research hotspots and future trends they transmitted in the field of immunology. We believe that this information can be useful in understanding the important information about COVID-19 provided by current immunology journals and the future research process.

## Methods

### Data sources and search strategies

**Figure 1:**
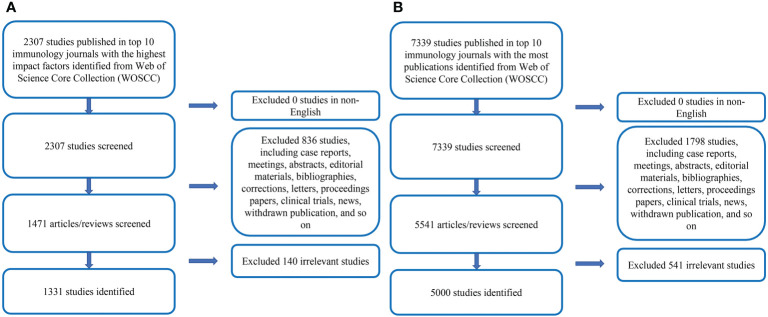
**(A)** Literature selection process for Part A. **(B)** Literature selection process for Part B.

We comprehensively searched the relevant literature on COVID-19 in the WOSCC. The search time span was from January 1, 2020, to July 31, 2022. The retrieval strategy was TS=(COVID-19 OR COVID 19 OR SARS-CoV-2 Infection OR Infection, SARS-CoV-2 OR SARS CoV 2 Infection OR SARS-CoV-2 Infections OR 2019 Novel Coronavirus Disease OR 2019 Novel Coronavirus Infection OR 2019-nCoV Disease OR 2019 nCoV Disease OR 2019-nCoV Diseases OR Disease, 2019-nCoV OR COVID-19 Virus Infection OR COVID 19 Virus Infection OR COVID-19 Virus Infections OR Infection, COVID-19 Virus OR Virus Infection, COVID-19 OR Coronavirus Disease 2019 OR Disease 2019, Coronavirus OR Coronavirus Disease-19 OR Coronavirus Disease 19 OR Severe Acute Respiratory Syndrome Coronavirus 2 Infection OR SARS Coronavirus 2 Infection OR COVID-19 Virus Disease OR COVID 19 Virus Disease OR COVID-19 Virus Diseases OR Disease, COVID-19 Virus OR Virus Disease, COVID-19 OR 2019-nCoV Infection OR 2019 nCoV Infection OR 2019-nCoV Infections OR Infection, 2019-nCoV OR COVID19 OR COVID-19 Pandemic OR COVID 19 Pandemic OR Pandemic, COVID-19 OR COVID-19 Pandemics). After excluding the literature that meets the language and article type requirements, further, evaluate the title and abstract articles to ascertain whether the literature meets the theme of COVID-19. For the uncertain literature, the full text was downloaded and assessed in more detail. **Figure 1** showed the research flow chart.

The journals of publication sources analyzed in this study were divided into two parts: In Part A, according to the latest JCR impact factor (IF) in 2021, we identified 10 immunology journals with the highest IF (**Table 1**). In Part B, we identified the 10 immunology journals with the most publications related to COVID-19 (**Table 1**).

**Table 1:** The journals included regarding COVID-19.

	Rank	Journals	Publications	Citations	H-index	IF(2021)	JCR (2021)	Country
Part A	1	CLINICAL INFECTIOUS DISEASES	790	41,680	82	20.999	Q1	USA
	2	EMERGING MICROBES INFECTIONS	237	12,138	48	19.568	Q1	ENGLAND
	3	BRAIN BEHAVIOR AND IMMUNITY	68	11,778	36	19.227	Q1	USA
	4	IMMUNITY	62	5,374	35	43.474	Q1	USA
	5	SCIENCE IMMUNOLOGY	57	5,418	35	30.63	Q1	USA
	6	NATURE IMMUNOLOGY	43	2,808	22	31.25	Q1	USA
	7	NATURE REVIEWS IMMUNOLOGY	33	6,234	22	108.555	Q1	ENGLAND
	8	CELLULAR MOLECULAR IMMUNOLOGY	26	1,589	13	22.096	Q1	CHINA
	9	TRENDS IN IMMUNOLOGY	11	579	7	19.709	Q1	USA
	10	ANNUAL REVIEW OF IMMUNOLOGY	4	11	2	32.481	Q1	USA
Part B	1	VACCINES	1,305	11,202	42	4.961	Q2	SWITZERLAND
	2	FRONTIERS IN IMMUNOLOGY	1,135	14,628	54	8.786	Q1	SWITZERLAND
	3	CLINICAL INFECTIOUS DISEASES	571	40,577	79	20.999	Q1	USA
	4	OPEN FORUM INFECTIOUS DISEASES	466	2,761	26	4.423	Q3	USA
	5	VACCINE	381	5,199	32	4.169	Q3	ENGLAND
	6	HUMAN VACCINES IMMUNOTHERAPEUTICS	340	3,278	23	4.526	Q3	USA
	7	JOURNAL OF INFECTIOUS DISEASES	284	7,510	45	7.759	Q1	USA
	8	EMERGING INFECTIOUS DISEASES	283	9,075	46	16.126	Q1	USA
	9	PEDIATRIC INFECTIOUS DISEASE JOURNAL	161	2,524	23	3.806	Q3	USA
	10	JOURNAL OF ALLERGY AND CLINICAL IMMUNOLOGY	74	5,715	37	14.29	Q1	USA

COVID-19, the coronavirus disease 2019; IF, impact factor; JCR, Journal Citation Reports.

### Bibliometric analysis

CiteSpace (6.1.3) was used to analyze the included literature, including co-citation analysis performed on countries/regions and institutions, dual-map overlay of citations, co-cited references analysis, and timeline view.

VOSviewer (1.6.16) was used to visualize the co-citation network of authors and journals, and the co-occurrence of keywords. In the visual map, different nodes represented authors, journals, keywords, etc. The node size indicated numbers or frequency. The thickness of the line represented the strength of the link. The colors of nodes represented different clusters.

In addition, R-Bibliometrix was used to analyze the theme evolution based on keywords over time, visualize the cooperation network between countries, and make a descriptive analysis of the publishing characteristics of journals. In addition, we used R software and R-Bibliometrix to generate the trend topics map based on the occurrence frequency of author keywords.

## Results

### General characteristics of publications

A total of 1,331 publications were included in Part A. The included publications had a total of 87,767 citations, with an average of 65.94 citations per paper, and an H-index of 132. A total of 5,000 publications were included in Part B. The included publications had a total of 102,469 citations, with an average of 20.49 citations per paper, and an H-index of 125.

### Countries/regions

#### Part A

**Figure 2:**
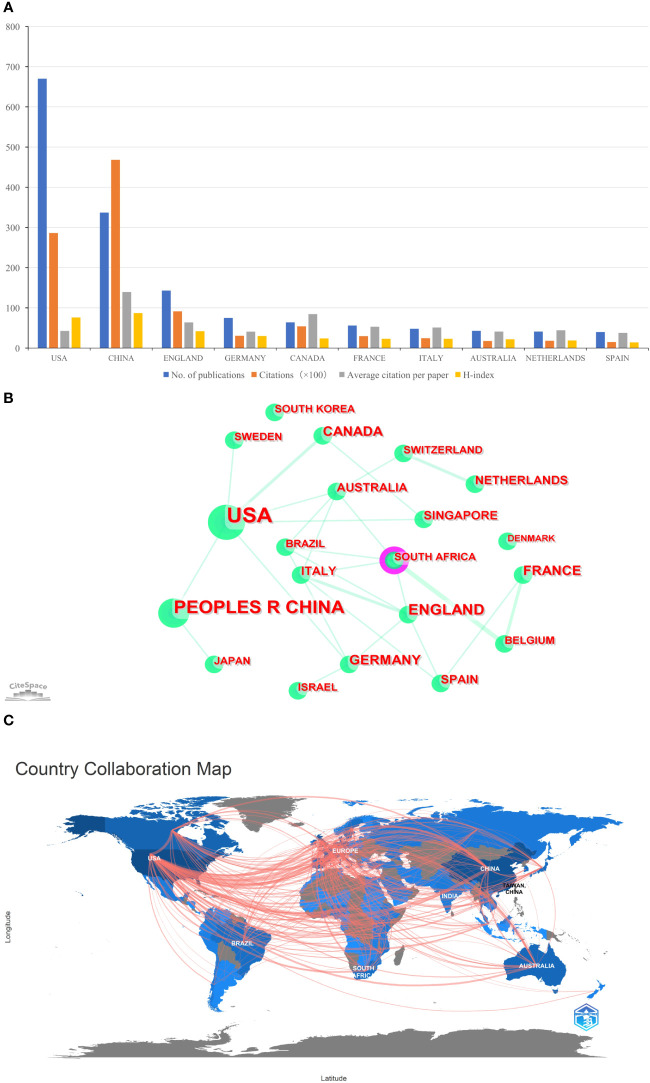
**(A)** The total publication number, total citations, average citation per paper, and H-index of the 10 most productive countries/regions of Part A. **(B)** The country collaboration network of Part A generated by Citespace. **(C)** The country collaboration of Part A plotted on the world map.

A total of 103 countries/regions contributed to the 1,331 publications included. The USA had the largest number of papers (n=670, accounting for 50.338% of the total; 28,625 citations, with an average of 42.72 citations per paper, and an H-index of 76), followed by China (n=336, accounting for 25.244%; 46,830 citations, with an average of 139.38 citations per paper, and an H-index of 87), and England (n=143, accounting for 10.744%; 9,137 citations, with an average of 63.9 citations per paper, and an H-index of 42) (**Figure 2A**). **Figures 2B, C** showed international cooperation among countries/regions. **Figure 2B** was generated by CiteSpace: The thickness of the lines between countries/regions showed the strength of cooperation. Of the 20 countries/regions with the most publications, the USA, Europe (England, Italy, Spain, and Germany), Australia, and South Africa cooperated closely with other countries/regions.

#### Part B

**Figure 3:**
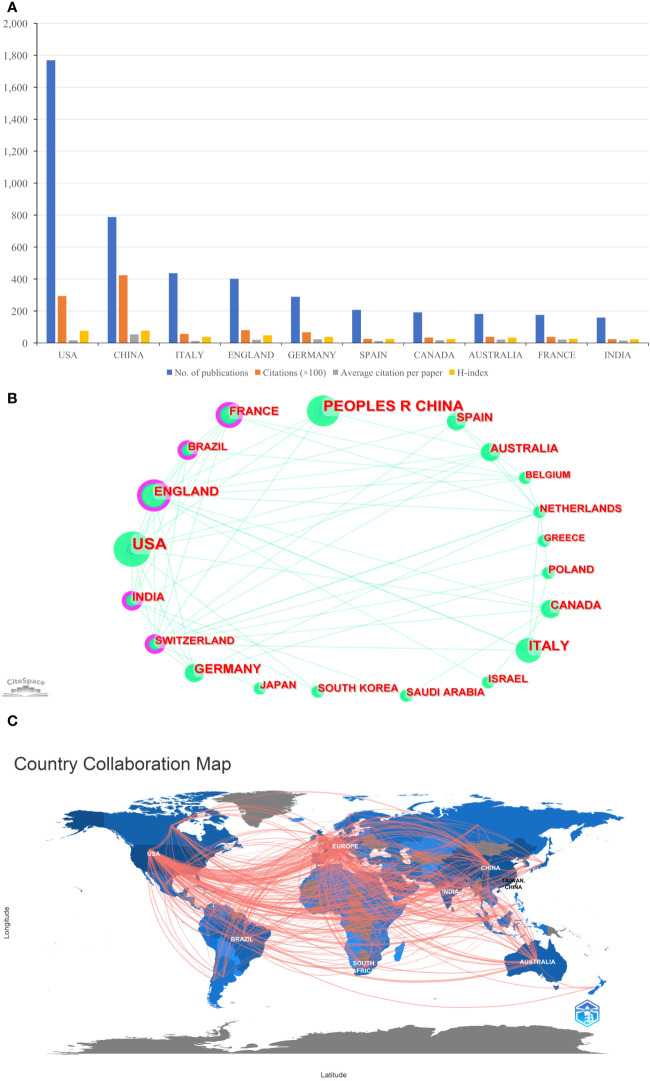
**(A)** The total publication number, total citations, average citation per paper, and H-index of the 10 most productive countries/regions of Part B. **(B)** The country collaboration network of Part B generated by Citespace. **(C)** The country collaboration of Part B plotted on the world map.

A total of 144 countries/regions contributed to the 5,000 publications included. The USA had the largest number of papers (n=1,769, accounting for 35.38% of the total; 29,376 citations, with an average of 16.61 citations per paper, and an H-index of 76), followed by China (n=788, accounting for 15.76%; 42,355 citations, with an average of 53.75 citations per paper, and an H-index of 77), and Italy (n=437, accounting for 8.74%; 5,660 citations, with an average of 12.95 citations per paper, and an H-index of 38) (**Figure 3A**). Although the total number of publications in England was fourth, total citations and the H-index were just behind the USA and China (**Figure 3A**). **Figures 3B, C** provided an illustration of international cooperation between countries/regions. Of the 20 countries/regions with the most publications, the USA and Europe had the closest cooperation with other countries/regions, notably England, Switzerland, and France. Furthermore, among developing countries, Brazil and India also have played a major role in national cooperation.

**Figures 2**, **3** showed that in the global cooperation against COVID-19, the trend of global cooperation has become very obvious. Developed countries such as the USA and Europe, and developing countries such as China and India remain the main contributors to global cooperation. However, developing countries were still a weak part of current global cooperation that could be linked to their research investment and weak infrastructure.

### Institutions

#### Part A

**Figure 4:**
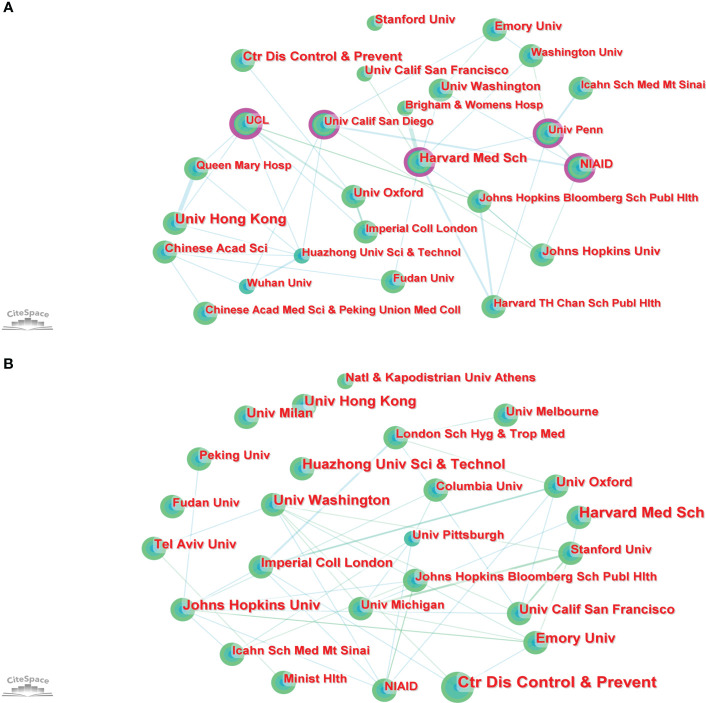
The collaboration network of institutions generated by Citespace of Part **A, B**.

In total, 2,717 institutions worldwide contributed to the 1,331 publications. CiteSpace generated a network visualization map of institutional collaboration, as shown in **Figure 4A**. The top 5 institutions with the most papers were the University of Hong Kong, the Center for Disease Control and Prevention, Harvard Medical School, Emory University, and the Chinese Academy of Sciences. The betweenness centrality (BC) value was an index to evaluate the importance of nodes in a collaborative network, and a BC value >0.1 was considered a vital node (36). Among the top 25 institutions with the most papers, institutions with BC values greater than 0.1 included University College London (UCL), National Institute of Allergy and Infectious Diseases (NIAID), Harvard Medical School, University California San Diego, and University of Pennsylvania. Of these 25 institutions, the USA had 15, China had 6, and England had 4 (**Table S1**).

#### Part B

In total, 7,037 institutions worldwide contributed to these 5,000 publications. CiteSpace generated a network visualization map of institutional collaboration, as shown in **Figure 4B**. The top 5 institutions with the most papers were the Center for Disease Control and Prevention, Harvard Medical School, University of Hong Kong, Emory University, and Johns Hopkins University. Of these 25 institutions, the USA had 13, China had 4, and England had 2 (**Table S1**).

### Authors and co-cited authors

#### Part A

**Figure 5:**
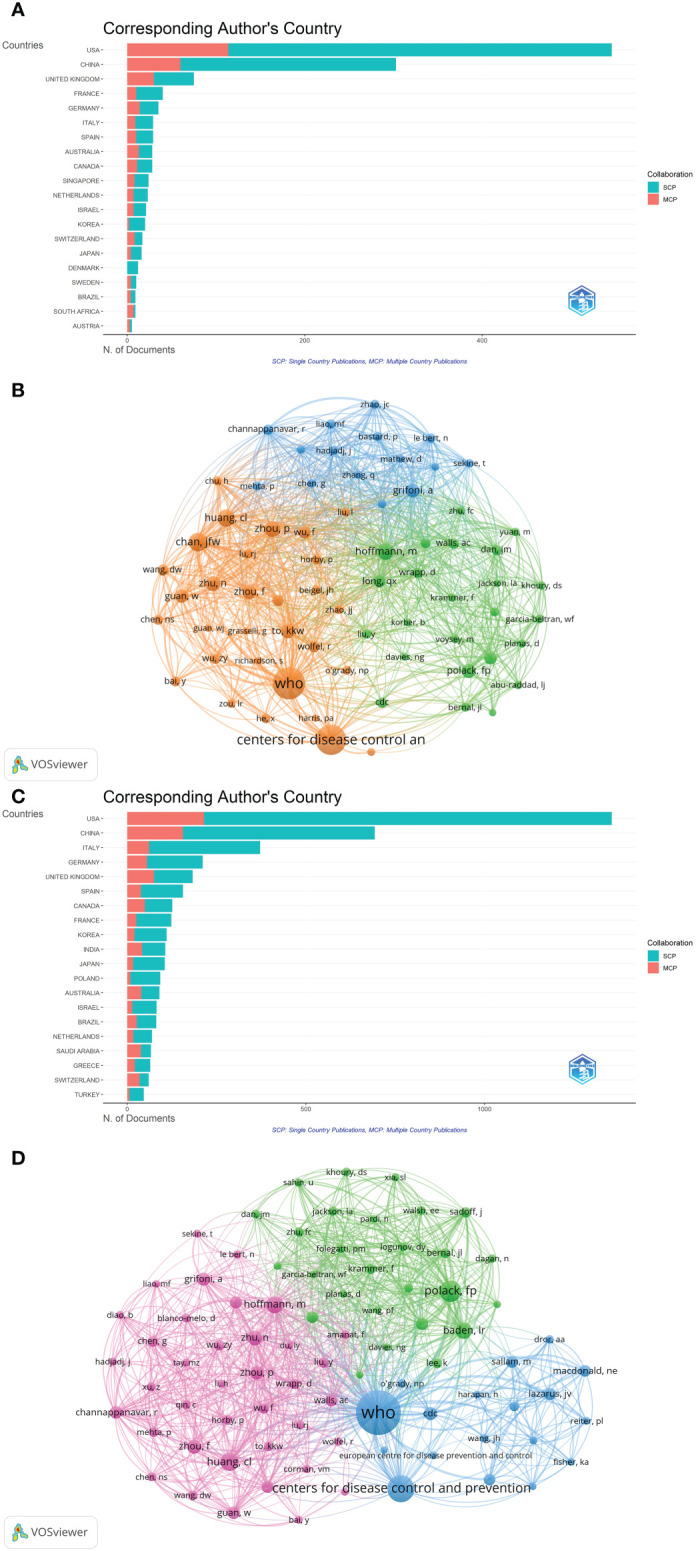
**(A)**The top 20 corresponding author’s countries of Part A. **(B)** The cluster density map of author co-authorship of Part A generated by VOSviewer. **(C)** The top 20 corresponding author’s countries of Part B. **(D)** The cluster density map of author co-authorship of Part B generated by VOSviewer.

A total of 14,793 authors contributed to the 1,331 publications, with an average of 11.11 authors per paper. Among the countries with the most corresponding authors of the papers, the USA ranked first, followed by China and the UK (**Figure 5A**). Among the top 12 authors with the most published papers, all were from China, 11 were from the University of Hong Kong, and 1 was from the National Institutes for Food and Drug Control (**Table 2**). Yuen Kwok Yung from the University of Hong Kong published the most papers with 4,802 citations and an H-index of 21, followed by To Kelvin Kai Wang from the University of Hong Kong, with 4,699 citations and an H-index of 20. By analyzing the co-citation network of authors, those who have been cited more than 40 times were defined as key researchers (**Figure 5B**): The connection represented the cooperation between authors, and the size of the circle represented the number of citations. Total link strength (TLS) indicates the impact of the authors’ published papers on other authors who participated in the studies. WHO had the most co-citations (n=448), followed by the Center for Disease Control and Prevention (n=398). The top 3 authors with the highest TLS were WHO, Chan Jasper Fuk Woo, and Grifoni Alba.

**Table 2:** The authors with the most publications.

	Rank	Authors	Country	Affiliation	Publications	Citations	H-index
Part A	1	Yuen Kwok Yung	CHINA	Univ Hong Kong	36	4802	21
	2	To Kelvin Kai Wang	CHINA	Univ Hong Kong	30	4699	20
	3	Chan Jasper Fuk Woo	CHINA	Univ Hong Kong	21	4194	14
	4	Chu Hin	CHINA	Univ Hong Kong	17	3046	12
	5	Hung Ivan Fan Ngai	CHINA	Univ Hong Kong	14	1670	12
	6	Chan kwok Hung	CHINA	Univ Hong Kong	13	2214	10
	7	Cheng Vincent Chi Chung	CHINA	Univ Hong Kong	12	1468	9
	8	Cai Jian Piao	CHINA	Univ Hong Kong	12	1392	8
	9	Cowling Benjamin J	CHINA	Univ Hong Kong	12	169	6
	10	Kok Kin Hang	CHINA	Univ Hong Kong	11	2689	8
	11	Yuan Shuofeng	CHINA	Univ Hong Kong	11	2640	8
	12	Huang Weijin	CHINA	Natl Inst Food & Drug Control	11	476	5
Part B	1	Yuen Kwok Yung	CHINA	Univ Hong Kong	22	2428	14
	2	To Kelvin Kai Wang	CHINA	Univ Hong Kong	20	2283	12
	3	Cowling Benjamin J	CHINA	Univ Hong Kong	19	220	6
	4	Dhama Kuldeep	INDIA	Indian Veterinary Research Institute	18	602	13
	5	Drosten Christian	GERMANY	Free University of Berlin	15	1245	9
	6	Hung Ivan Fan Ngai	CHINA	Univ Hong Kong	14	1244	8
	7	Tate Jacqueline E	USA	Centers for Disease Control & Prevention	14	161	8
	8	Kirking Hannah L	USA	Centers for Disease Control & Prevention	13	153	7
	9	Chan kwok Hung	CHINA	Univ Hong Kong	12	1816	8
	10	Thornburg Natalie J	USA	Centers for Disease Control & Prevention	12	46	7

#### Part B

A total of 35,394 authors contributed to the 5,000 publications, with an average of 7.08 authors per paper. Among the countries with the most corresponding authors of the papers, the USA ranked first, China ranked second, and Italy ranked third (**Figure 5C**). Among the top 10 authors with the most published papers, 5 came from China, all from the University of Hong Kong, and 3 from the USA, all from the Center for Disease Control and Prevention (**Table 2**). Yuen Kwok Yung from the University of Hong Kong published the most papers, with 2,428 citations and an H-index of 14, followed by To Kelvin Kai Wang from the University of Hong Kong, with 2,283 citations and an H-index of 12. Although Dhama Kuldeep from Indian Veterinary Research Institute only ranked fourth in the number of articles, the citations ranked third (n=602) and H-index ranked second (n=13) of the publications. By analyzing the co-citation network of authors, those who have been cited more than 115 times were defined as key researchers (**Figure 5D**). WHO had the most co-citations (n=2,247), and the top 3 authors with the highest TLS were WHO, Polack Fernando P, and Hoffmann Markus.

### Journals

#### Part A

**Figure 6:**
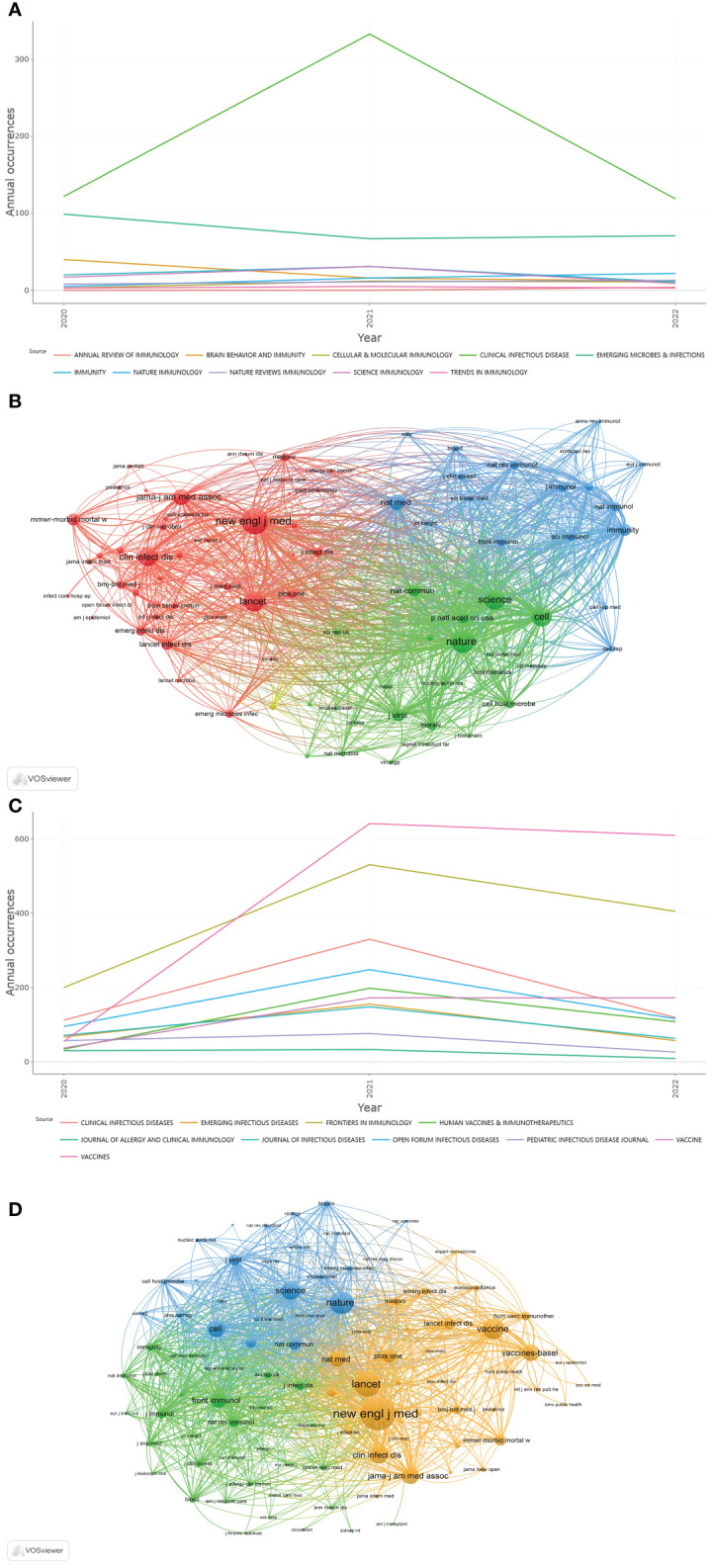
**(A)** Annual occurrences of the 10 journals of Part A. **(B)** The network visualization diagram of journal co-citation analysis of Part A generated by VOSviewer. **(C)** Annual occurrences of the 10 journals of Part B. **(D)** The network visualization diagram of journal co-citation analysis of Part B generated by VOSviewer.

Among the top 10 immunology journals with the highest IF, *CLINICAL INFECTIOUS DISEASES* had the most publications (n=790), followed by *EMERGING MICROBES INFECTIONS* and *BRAIN BEHAVIOR AND IMMUNITY* (n=237 and 68, respectively) (**Table 1**). Of these 10 journals, 7 were made available in the USA, and 2 were published in England. *CLINICAL INFECTIOUS DISEASES* had the highest total citations (n=41,680) and H-index (n=82), followed by *EMERGING MICROBES INFECTIONS* (12,138 total citations and 48 of H-index). According to the latest JCR division in 2021, all 10 journals were in the JCR Q1, of which *NATURE REVIEWS IMMUNOLOGY* had the highest IF (108.555), followed by *IMMUNITY* (43.474). In addition, the annual occurrences of the 10 journals were generated by R-Bibliometrix to understand more specifically the trend of the publication numbers of these journals in different years (**Figure 6A**). It could be noted that from 2020 to 2021, the number of publications by *CLINICAL INFECTIOUS DISEASES* increased dramatically. As shown in **Figure 6B**, the network visualization diagram of journal co-citation analysis was created by VOSviewer. Only visually cited journals at least 70 times. Among the 84 journals that met the standard, the top 5 journals commonly cited were *NEW ENGLAND JOURNAL OF MEDICINE* (2,610 times), *NATURE* (2,098 times), *SCIENCE* (1,683 times), *LANCET* (1,633 times), and *CELL* (1,599 times).

#### Part B

Among the 10 most published immunology journals, *VACCINES* had the largest number of publications (n=1,305), followed by *FRONTIERS IN IMMUNOLOGY* and *CLINICAL INFECTIOUS DISEASES* (n=1,135 and 571, respectively) (**Table 1**). Of these 10 journals, 7 were made available in the USA, and 2 were published in Switzerland. *CLINICAL INFECTIOUS DISEASES* had the highest total citations (n=40,577) and H-index (n=79), followed by *FRONTIERS IN IMMUNOLOGY* (14,628 total citations with an H-index of 54). 5 journals were in the JCR Q1, of which *CLINICAL INFECTIOUS DISEASES* had the highest IF (20.999), followed by *EMERGING INFECTIOUS DISEASES* (16.126). From the annual occurrences of the 10 journals (**Figure 6C**), it could be found that from 2020, the publication volume of *VACCINES* has increased the fastest. The network visualization diagram of journal co-citation analysis visualizes only journals that have been cited at least 300 times (**Figure 6D**). Among the 93 journals that met the standard, the top 5 journals commonly cited were *NEW ENGLAND JOURNAL OF MEDICINE* (8,528 times), *LANCET* (5,894 times), *NATURE* (5,234 times), *VACCINE* (4,650 times), and *SCIENCE* (4,010 times).

### Dual-map overlays

**Figure 7:**
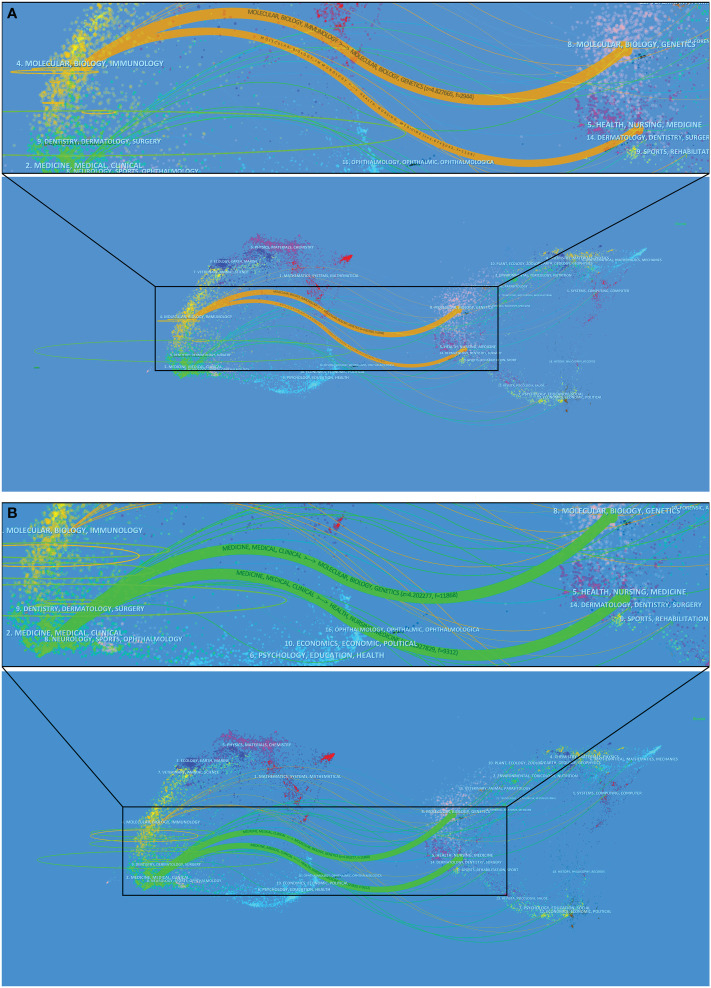
The dual-map overlay of the journals of Part **A, B**.

The superposition of dual-map overlays revealed the overall scientific contribution. The left side was the citing journal, the right side was the cited journal, and the colored line path represented the citation relationship, indicating the citation trajectory and knowledge flow of knowledge (37). The result of Part A indicated, that the published articles related to COVID-19 were mainly focused on journals in the field of molecular, biology, and immunology, whereas most of the cited articles were published in journals in the field of molecular, biology, genetics, and health, nursing, medicine (**Figure 7A**). The result of Part B indicated, the published articles related to COVID-19 were mainly focused on journals in the field of medicine, medical, and clinical, whereas most of the cited articles were published in journals in the field of molecular, biology, genetics, and health, nursing, medicine (**Figure 7B**).

### Co-cited references

**Figure 8:**
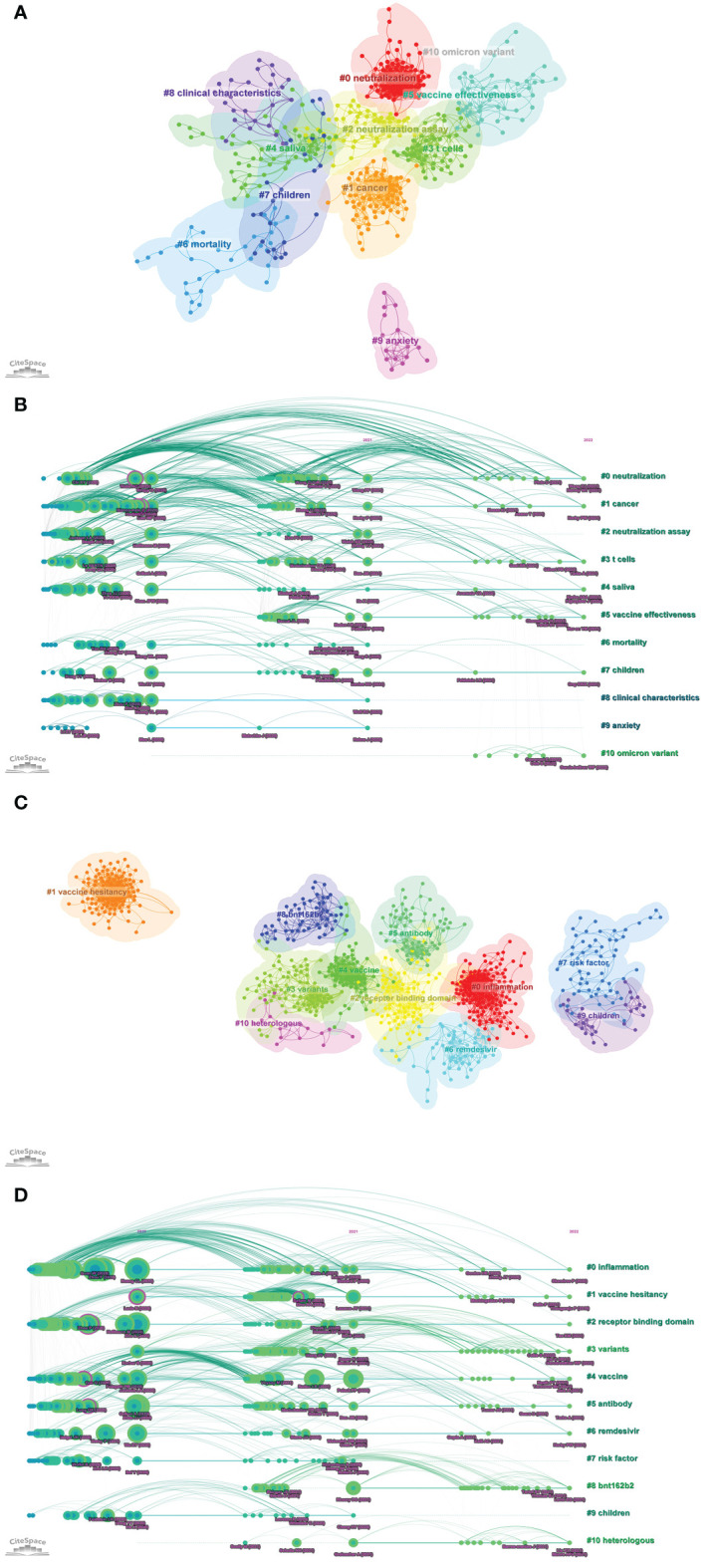
**(A)** The visualization network of co-cited references of Part A generated by Citespace. **(B)** The timeline view map of reference co-citation analysis of Part A generated by CiteSpace. **(C)** The visualization network of co-cited references of Part B generated by Citespace. **(D)** The timeline view map of reference co-citation analysis of Part B generated by CiteSpace.

Top 10 highly co-cited references of Part A and Part B were summarized in **Supplementary Table S2**, respectively. Part A: The analysis results of co-cited references by CiteSpace were shown in **Figure 8A**. In the visualization network of co-cited references, all nodes representing the references were clustered into 11 specific clusters with the highest K values, and further formed the timeline view of these 11 clusters (**Figure 8B**). The modularity was 0.6214, and the mean silhouette was 0.8706, which reflected the rationality of the clustering method. All these clusters were summarized and ordered by the number of co-cited references: the first cluster was “#0 neutralization”, followed by “#1 cancer”, “#2 neutralization assay”, and “#3 T cells”. Part B: all nodes representing the references were clustered into 11 specific clusters with the highest K values (**Figure 8C**), and further formed the timeline view of these 11 clusters (**Figure 8D**). The modularity was 0.7476, and the mean silhouette was 0.9096. All these clusters were summarized and ordered by the number of co-cited references: the first cluster was “#0 inflammation”, followed by “#1 vaccine hesitancy”, “#2 receptor binding domain”, and “#3 variants”.

### Keywords

#### Part A

**Figure 9:**
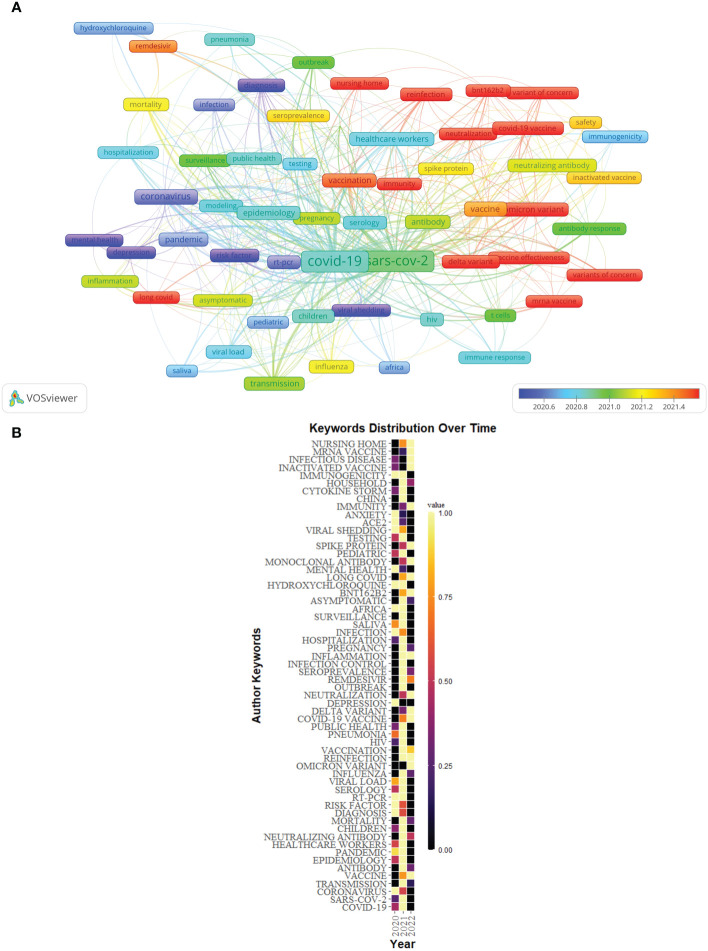
**(A)** Overlay visualization of keywords of Part A generated by VOSviewer. **(B)** The distribution map of high-frequency keywords over time of Part A generated by R software.

**Figure 9A** showed the overlay visualization of author keywords generated by VOSviewer. Among 2,097 keywords, the frequency of occurrence was set to at least 9, and finally, 62 keywords were incorporated into the analysis. All these keywords were marked with different colors, which could reflect the research hotspots in different periods. Older keywords appeared in blue, while red represented more recent keywords. For example, keywords such as “depression”, “risk factor”, “mental health”, and “viral shedding” were the main topics in the early stage, and the keywords of “long COVID”、”immunity”, “neutralization”, “variant of concern”, “Delta variant”, “Omicron variant”, “BNT162b2”, “vaccine effectiveness”, and “mRNA vaccine” were hot topics.

In addition, the distribution map of the top 60 high-frequency keywords over time was conducted by R software (**Figure 9B**), in which each cell represented the occurrence frequency of a keyword in a year, and the corresponding value was formed after standardizing these occurrence frequencies (0–1). The value of the black cell was the smallest, which represented the lowest occurrence frequency of the keyword this year, with the change of color, the value of the yellow cell was the biggest, and its corresponding keywords appeared the most frequently this year. For example, “depression” appeared more frequently in 2020, however, the frequency in 2021 and 2022 (as of July 31, 2022) was low.

**Figure 10:**
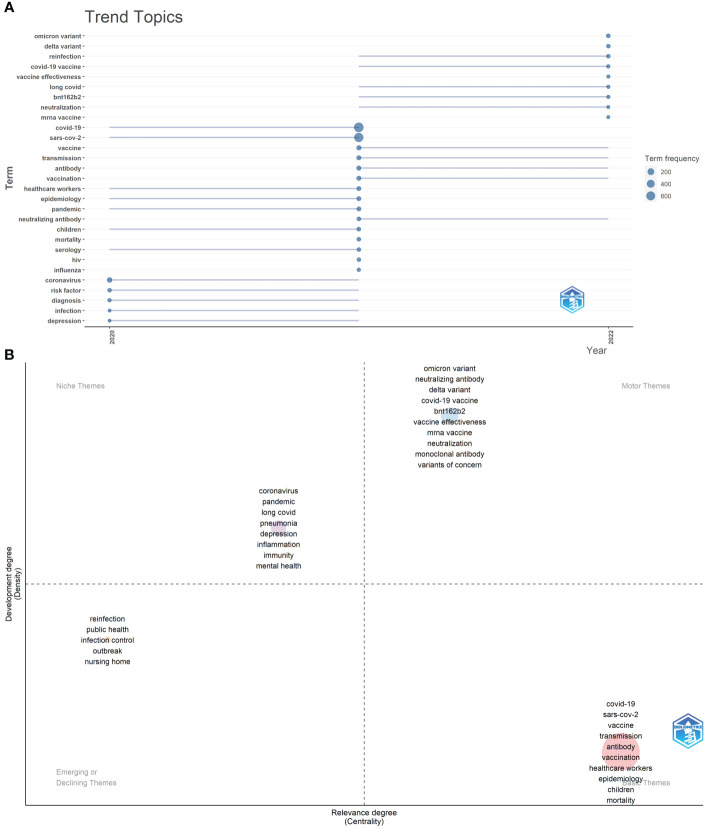
**(A)** Trend topics of Part A. The X-axis represents the year, while the Y-axis is the cumulative occurrences of the keywords. **(B)** The keywords thematic map of Part A generated by R-Bibliometrix.

The trend topic analysis was an important mapping tool that helped to portray the seed of trend integration rooted in the previous stream (38). The trend topics map was generated by R-Bibliometrix based on the occurrence frequency of author keywords and set word minimum frequency=12 and the number of words per year=15 (**Figure 10A**). The results showed that “reinfection”, “long COVID”, “BNT162b2” and “neutralization” began to appear in the immunology field of COVID-19 in 2021, “Delta variant”, “Omicron variant”, “vaccine effectiveness”, and “mRNA vaccine” had a higher frequency of occurrence in 2022 (as of July 31, 2022).

Finally, we conducted the keywords thematic map by R-Bibliometrix (**Figure 10B**), and a total of 350 keywords were examined where a minimum cluster frequency was 7 and the number of labels for each cluster was 10. The upper right quadrant (motor theme), which was characterized by a high density and centrality showed probably the well-developed and important themes for the structuring of the COVID-19 research field, including “Delta variant”, “Omicron variant”, “neutralizing antibody”, “neutralization”, “vaccine effectiveness”, “BNT162b2”, “mRNA vaccine”, “monoclonal antibody”, and “variant of concern”. In the upper-left quadrant (niche theme), it was possible to find the themes “depression”, “mental health”, “pandemic”, “long COVID”, “pneumonia”, “immunity”, and “inflammation” as major keywords. The cluster in the third quadrant (emerging or declining theme) was characterized by low centrality and density, which means that it was weakly developed and marginal, including “reinfection”, “public health”, “infection control”, “outbreak”, and “nursing home”. The fourth quadrant (basic themes) contained “COVID-19”, “SARS-CoV-2”, “vaccine”, “transmission”, “antibody”, “vaccination”, “healthcare workers”, “epidemiology”, “children”, and “mortality” as the major themes. They were concerned with general topics that were transversal to different research areas in the field.

#### Part B

**Figure 11:**
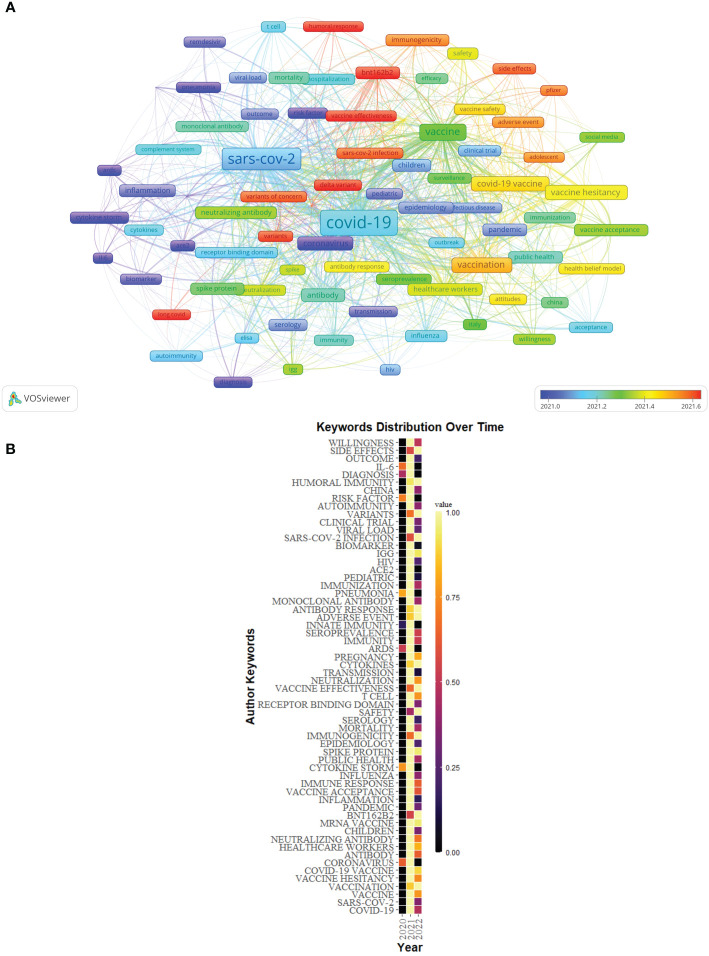
**(A)** Overlay visualization of keywords of Part B generated by VOSviewer. **(B)** The distribution map of high-frequency keywords over time of Part B generated by R software.

84 author keywords with at least 25 occurrences were extracted from 5,000 publications to create an overlay visualization (**Figure 11A**). Keywords such as “diagnosis”, “risk factor”, “coronavirus”, and “IL-6” were the main topics in the early stage, and the keywords of “long COVID”, “Delta variant”, “Omicron variant”, “BNT162b2”, “vaccine effectiveness”, and “humoral response” were hot topics.

The distribution map of the top 60 high-frequency keywords over time also was conducted by R software (**Figure 11B**).

**Figure 12:**
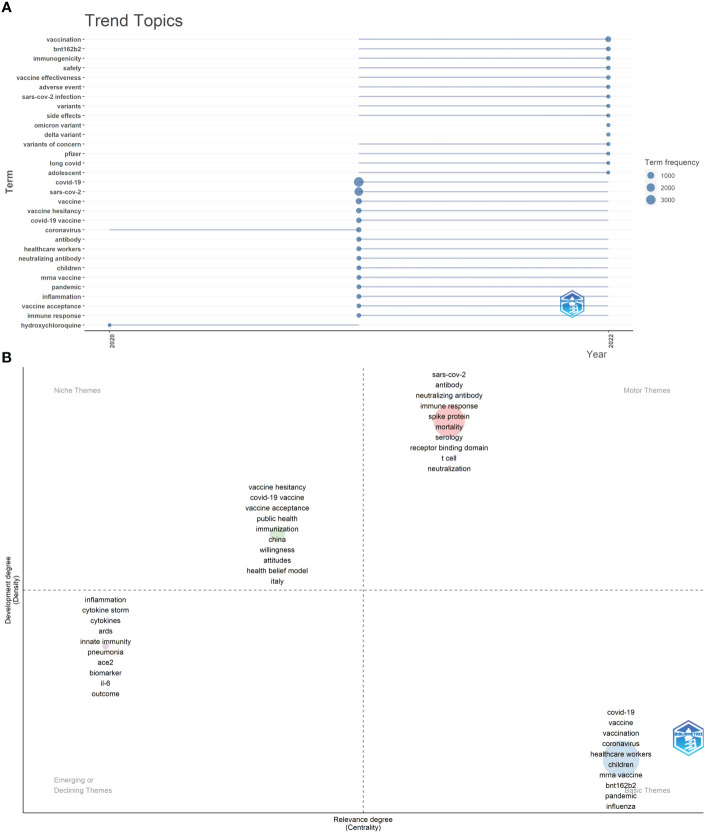
**(A)** Trend topics of Part B. The X-axis represents the year, while the Y-axis is the cumulative occurrences of the keywords. **(B)** The keywords thematic map of Part B generated by R-Bibliometrix.

We also plotted the trend topics map (word minimum frequency=20, number of words per year=15) (**Figure 12A**). The results showed that “vaccination”, “BNT162b2”, “immunogenicity”, “safety”, “vaccine effectiveness”, “adverse event”, “side effects”, “variants of concern”, “long COVID”, and “Pfizer” began to appear in the immunology field of COVID-19 in 2021, “Delta variant” and “Omicron variant” began to appear in the immunology field of COVID-19 in 2022. The above topics had a higher frequency of occurrence in 2022 (as of July 31, 2022).

In the keywords thematic map (**Figure 12B**), a total of 450 keywords were examined where a minimum cluster frequency was 5 and the number of labels for each cluster was 10. The motor theme included “SARS-CoV-2”, “antibody”, “neutralizing antibody”, “immune response”, “spike protein”, “mortality”, “serology”, “receptor binding domain”, “T cell”, and “neutralization”. The niche theme includes “vaccine hesitancy”, “COVID-19 vaccine”, “vaccine acceptance”, “public health”, “immunization”, “China”, “willingness”, “attitudes”, “health belief model”, and “Italy”. The emerging or declining theme included “inflammation”, “cytokine storm”, “cytokines”, “ARDS”, “innate immunity”, “pneumonia”, “ACE2”, “biomarker”, “IL-6”, and “outcome”. The basic themes contained “COVID-19”, “vaccine”, “vaccination”, “coronavirus”, “healthcare workers”, “children”, “mRNA vaccine”, “BNT162b2”, “pandemic”, and “influenza” as the major themes.

## Discussion

**Figure 13:**
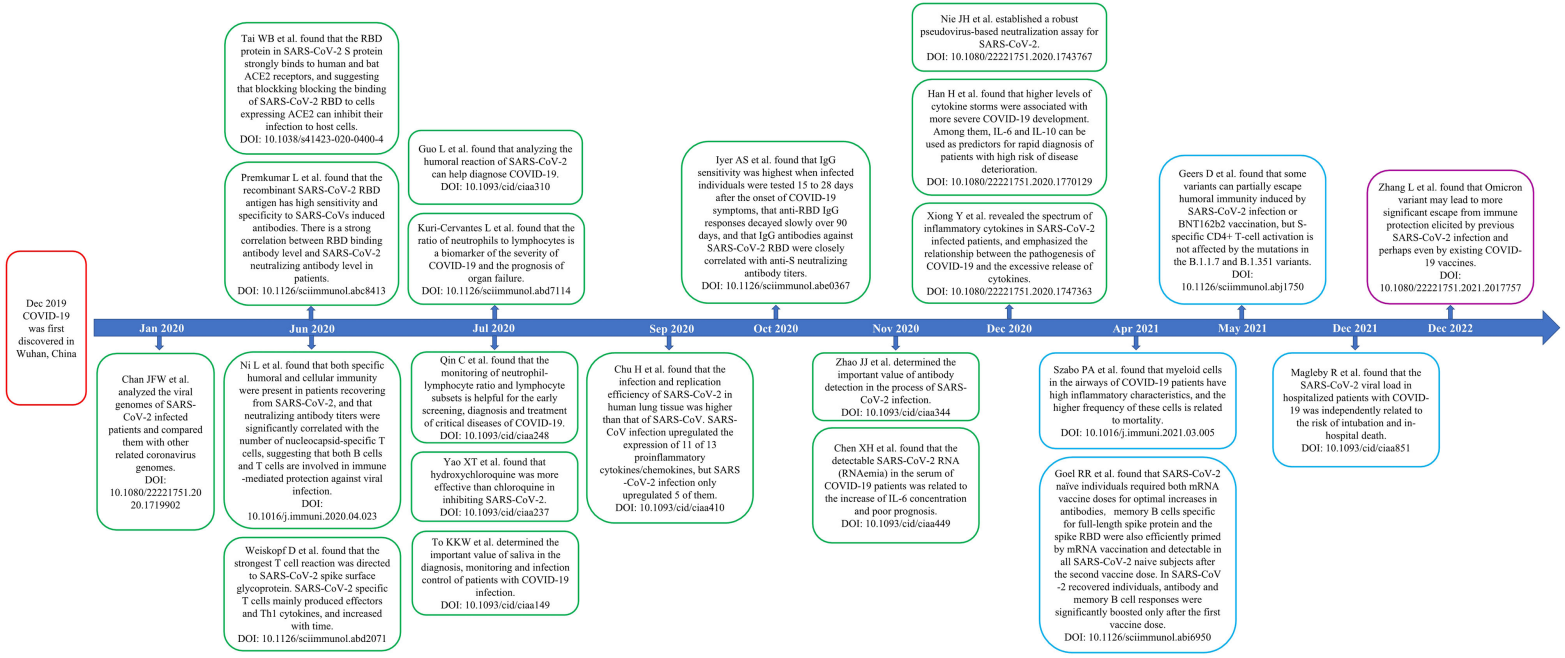
Timeline of some landmark papers. Abbreviation: COVID-19, the coronavirus disease 2019; RBD, receptor binding domain; ACE2, angiotensin-converting enzyme II.

In this study, we used CiteSpace, R-Bibliometrix, and VOSviewer to perform a bibliometric and visual analysis of COVID-19-related papers published in major immunology journals between 2020 and 2022 to sort out the research status and hotspots of the publications and forecast future research trends. In addition, we identified some landmark papers (**Figure 13**). The journals of publication source analyzed in this study were divided into two parts: Part A, according to the latest JCR IF in 2021, we identified 10 immunology journals with the highest IF. In Part B, we identified the 10 immunology journals with the most publications related to COVID-19.

The number of publications in a country are a major indicator of a country’s output. The results of Part A and B both showed that the USA and China were the two countries with the most papers on COVID-19 published in major immunological journals. Part B showed that Italy was the third country in terms of publications, but although the total number of publications in England ranked fourth, its total citations and H-index were second only to the USA and China. It was worth noting that the results of Part A showed that although the total number of publications in the USA was higher than that in China, China ranked first in terms of total citations, average citations, and H-index, which showed that there were important highly cited articles in 336 papers published in journals with high impact factors in China, of which 10 papers (39–48) have been cited more than 800 times and 6 papers (39–44) have been cited more than 1,000 times. Among these highly cited papers, Zhao et al. (39) published a paper in *CLINICAL INFECTIOUS DISEASES* with a high number of citations (n=11,050), in which they examined total antibodies, IgM, and IgG to SARS-CoV-2 in plasma samples from 173 SARS-CoV-2-infected patients, and the dynamics of antibodies with disease progression were analyzed. Combining the results of Part A and B, we found that in the global cooperation against COVID-19, the trend of global cooperation has become very obvious, this was due to the global pandemic of COVID-19, and almost all countries in the world were under the threat of COVID-19. Currently, developed countries such as the USA and European countries, as well as major developing countries such as China, India, and South Africa, were still major contributors to global cooperation, however, developing countries were still the weak part of the current global cooperation, which might be related to their weak scientific research input and infrastructure.

At the institution level, the results of Part A showed that among the top 25 institutions with the most publications, UCL, NIAID, Harvard Medical School, University California San Diego, and University of Pennsylvania all had BC values greater than 0.1, however, the results of Part B showed that the BC values of UCL, NIAID, Harvard Medical School, University California San Diego, and University of Pennsylvania were 0.06, 0.05, 0.08, 0.00, and 0.07, respectively. In the knowledge map generated by CiteSpace, the BC value of a node referred to the number of all the shortest paths that passed through the node in the network and was a measure of the connection role of the node in the overall network. For this result, it might be because the papers published in part B involved far more institutions than in Part A, which led to the fact that the institutions that played an important role in Part A seemed less important in Part B. After all, more institutions shared the connection role. However, these institutions still played an important role in international cooperation in this field. From the results of Part A and B, it could be seen that although some institutions cooperated with institutions in other countries, more of them cooperated with institutions in their own countries. For example, Harvard Medical School has carried out some cooperation with the Fudan University of China, but the USA institutions such as Brigham and Women’s Hospital, University of California, San Francisco, University of Washington, University of Pennsylvania, and Johns Hopkins Bloomberg School of Public Health had the most cooperation with them (**Figure 4A**). Although the benign development trend of global institutional cooperation has been shown in this field, cooperation, achievement exchange and information sharing between institutions in different countries still need to be strengthened. This was also a reminder for immunology journals, that was, to promote the publication of high-level papers with multi-center cooperation.

The results of Part A showed that among the 12 most productive authors, 11 were from the University of Hong Kong. The results of Part B showed that among the 10 most productive authors, 5 were from the University of Hong Kong and 3 were from the Centers for Disease Control & Prevention. Combining the results of Part A and B, it was found that Yuen Kwok Yung from the University of Hong Kong was the most important author in terms of the number of publications and citations, and the H-index. Among the papers contained in Part A, two papers published by Yuen Kwok Yung were cited more than 1,000 times: An article published in *EMERGING MICROBES & INFECTIONS* was cited 1,478 times. The authors conducted bioinformatics analysis on the viral genome of SARS-CoV-2 infected patients and compared them with other related coronavirus genomes. They found that the genome of SARS-CoV-2 had 89% nucleotide identity with bat SARS-like-CoVZXC21 and 82% with that of human SARS-CoV. The phylogenetic trees of their orf1a/b, Spike, Envelope, Membrane, and Nucleoprotein also clustered closely with those of the bat, civet, and human SARS coronaviruses (41). The other paper published in *CLINICAL INFECTIOUS DISEASES* confirmed that saliva might become a non-invasive sample type for SARS-CoV-2 diagnosis and viral load monitoring, and the risk of hospital transmission of the virus could be reduced (44). The number of citations for this paper was 1,023. In addition, this paper was also the highest cited paper among the author’s papers included in Part B.

As for the author co-citation analysis, as a group author, WHO was the author with the most co-citations (n=448 and 2,247, respectively) as shown by the results of Part A and B. Among the papers published by WHO on COVID-19, 2 articles have been cited more than 1,000 times: 1 paper was published in the *NEW ENGLAND JOURNAL OF MEDICINE* with 1,279 citations reporting the effect of four antiviral drugs (remdesivir, hydroxychloroquine, lopinavir, and interferon beta-1a) on overall mortality, ventilation initiation, and length of hospital stay of COVID-19 hospitalization patients (49). Another META analysis published in *JAMA* with 1,012 citations found that the application of systemic corticosteroids was associated with lower 28-day all-cause mortality in COVID-19 critical patients compared with routine care or placebo groups (50). Undoubtedly, since WHO declared COVID-19 as a public health emergency of international concern on January 30, 2020, which has made the most important contribution to the global fight against COVID-19: WHO rapidly scaled up its response including through its 149 country offices to support Member States prepare for and respond to the COVID-19 pandemic (51). Moreover, WHO made great efforts and contributions in many aspects such as carrying out clinical research on COVID-19 (49), developing COVID-19 vaccine and formulating international standards for antibody response of COVID-19 vaccine (52, 53), formulating COVID-19 discharge guidelines (54) and WHO living guideline for drugs to prevent COVID-19 (55), and formulating regional and national coordination mechanisms for managing COVID-19 pandemic (56).

Analyzing the characteristics of international peer-reviewed journals was helpful to understand the current trend, which was directly reflected in helping scholars understand the major immunology journals related to the field of COVID-19, and select the most appropriate published journals for their research. The results of Part A showed that among the top 10 immunology journals with the highest IF, *CLINICAL INFECTIOUS DISEASES* had the most publications (n=790), followed by *EMERGING MICROBES INFECTIONS* (n=237). Among the articles on COVID-19 published on *CLINICAL INFECTIOUS DISEASES*, the paper published by Zhao et al. (39) was the most cited. It was worth mentioning that among the papers published on COVID-19, the number of citations of this paper ranked fifth, second only to two papers published in *LANCET (* 57, 58), and two papers published in *NEW ENGLAND JOURNAL OF MEDICINE (* 59, 60). Moreover, according to Essential Science Indicators of WOS, during the 2022 March/April period, the citation frequency of this paper has entered the top 0.1% in the academic field of immunology. It could be observed in **Figure 6A** that as an immunology journal with high IF, the publications on COVID-19 published by *CLINICAL INFECTIOUS DISEASES* from 2020 to 2021 ranked first in terms of total publications and growth rate. This indicated that in the field of immunology journals, this journal was the regional core and popular journal in this field, and made great contributions to the dissemination of COVID-19 immunology-related information. Results of Part B showed that *VACCINES* had the largest number of publications (n=1,305), followed by *FRONTIERS IN IMMUNOLOGY* (n=1,135). The number of papers on COVID-19 published by *VACCINES* increased from 55 in 2020 to 641 in 2021 and 609 by July 2022. The highest cited papers (529 times) on COVID-19 published in this journal reviewed the latest assessment of the global COVID-19 vaccination acceptance rate (61). A paper published in *FRONTIERS IN IMMUNOLOGY*, which was cited 1,183 times, analyzed the number and functional status of T cells in patients with COVID-19. The authors found that patients with COVID-19 had significantly reduced T cell counts, surviving T cells appear to be functionally exhausted, and non-ICU patients with total T cell counts below 800/μL might still require urgent intervention (62). According to Essential Science Indicators of WOS, this paper has been cited among the best 0.1% in the academic field of immunology. From the perspective of the number of publications and citations, and H-index, *CLINICAL INFECTIOUS DISEASES*, and *FRONTIERS IN IMMUNOLOGY* were the most essential immunology journals in the field of COVID-19-related research (**Table 1**). Combining the results of Part A and B, *NEW ENGLAND JOURNAL OF MEDICINE*, *LANCET*, *NATURE*, *VACCINE*, *SCIENCE*, and *CELL* were the most co-cited journals. Unlike several other journals with high worldwide impact, *VACCINE* had a lower IF (4.169). However, we found that among the papers on COVID-19 published in *VACCINE*, 6 papers were cited more than 150 times (63–68). In conclusion, the research results related to COVID-19 published in these journals were more likely to be cited and received more attention. Moreover, it was necessary to pay attention to the published papers in these journals to get the latest progress in the field of COVID-19.

Reference co-citation analysis and keyword co-occurrence analysis were helpful to reveal the main research directions, hotspots, and evolutionary processes in this field (69).

The timeline view helped to understand the evolutionary trajectory of topics in the field (70). As could be seen from the timeline view of Part A, from 2021 to 2022, the three clusters “#0 neutralization”, “#3 T cells”, and “#5 vaccine effectiveness” had the highest frequency of citing references. Similarly, the timeline view of Part B showed that the three clusters “#3 variants”, “#8 BNT162b2”, and “#10 heterologous” had the highest frequency of citing references. These results suggested that the above topics were the current research hotspots in the immunology field of COVID-19.

The overlay visualization of author keywords by VOSviewer indicated that the results of Part A showed that “long COVID”, “immunity”, “neutralization”, “variant of concern”, “Delta variant”, “Omicron variant”, “BNT162b2”, “vaccine effectiveness”, and “mRNA vaccine” were relatively new topics in Part A. The results of Part B showed that the keywords “long COVID”, “Delta variant”, “Omicron variant”, “BNT162b2”, “vaccine effectiveness”, and “humoral response” were relatively new topics. Combining the results of Part A and B, the current research hotspots mainly focused on SARS-CoV-2 variant-related topics, such as “Delta variant” and “Omicron variant”, and immunization or vaccine-related topics for SARS-CoV-2, such as “BNT162b2”, “vaccine effectiveness”, “immunity”, “neutralization”, “mRNA vaccine”, and “humoral response”, and the topic “long COVID”.

We also took other methods to analyze the keywords. The trend topics map of Part A showed that “Delta variant”, “Omicron variant”, “vaccine effectiveness”, “mRNA vaccine”, “reinfection”, “long COVID”, “BNT162b2”, and “neutralization” were the topics worth paying attention to in 2021-2022. The results of Part B showed that “Delta variant”, “Omicron variant”, “vaccination”, “BNT162b2”, “immunogenicity”, “safety”, “vaccine effectiveness”, “adverse event”, “side effects”, “variants of concern”, and “long COVID” were the most noteworthy topics in 2021-2022. Combining the results of Part A and B, the topics worthy of attention at present mainly focused on the related topics of the SARS-CoV-2 variant, such as “Delta variant” and “Omicron variant”, the immune-related topics against SARS-CoV-2, such as “neutralization”, “reinfection”, “immunogenicity”, the vaccine-related topics such as “BNT162b2”, “vaccine effectiveness”, “mRNA vaccine”, “safety”, “adverse event”, and “side effects”, and “long COVID”.

Finally, we focused on the motor theme in the keywords thematic map. The results of Part A indicated that “Delta variant”, “Omicron variant”, “neutralizing antibody”, “neutralization”, “vaccine effectiveness”, “BNT162b2”, “mRNA vaccine”, “monoclonal antibody”, and “variant of concern” were well-developed and important themes. The results of Part B showed that “antibody”, “neutralizing antibody”, “immune response”, “spike protein”, “mortality”, “serology”, “receptor binding domain”, “T cell”, and “neutralization” were well-developed and important themes. In addition, “cytokine storm” in the third quarter (emerging or declining theme), as a new theme, is also worthy of attention.

Different software and research methods had their advantages and could complement each other. In this study, we used VOSviewer, R software, and R-Bibliometrix to analyze the keywords, using keyword overlay visualization, trend topics map, distribution map of high-frequency keywords, and keywords thematic map to show the topic evolution and hotspots of keywords as fully and objectively as possible. Based on the above results, we found that the topics of common concern among the 10 immunology journals with high IF and the 10 most published immunology journals were mainly focused on the SARS-CoV-2 variant-related topics, such as “Delta variant” and “Omicron variant”, immune-related topics against SARS-CoV-2, such as “neutralization/neutralizing antibody”, “T cell”, vaccine-related topics, such as “BNT162b2”, “vaccine effectiveness”, “mRNA vaccine”, and “safety”, and the topic “long COVID”.

### SARS-CoV-2 variants

As the most prevalent variant of concern in 2021 (71), the Delta variant was first detected in India in October 2020 and reported in over 80 countries on 20 June 2021 (72). Enhanced transmission of Delta variants is associated with key mutations in spike proteins such as P681R, L452R, D614G, and T478K. Although vaccines such as ChAdOx1 nCoV-19 and BNT162b2, which are widely used in different countries, are resistant to Delta variants, their efficiency is still much lower. There is imperative to reveal the molecular, immune, and toxic characteristics of this variant to effectively eradicate the Delta variant. The latest researches suggest that chimeric spike mRNA vaccines may be the direction to overcome the Delta variant (73, 74).

Since the first case of Omicron variant infection was discovered in South Africa on November 24, 2021 (75), this variant has rapidly spread to many countries and regions around the world. All the Omicron subvariants share eleven common mutations in the RBD of the spike protein, which is responsible for enhanced transmission, evasion, antibody neutralization, and RNA expression (76). Omicron variant can evade neutralizing antibody (NA) and weaken existing COVID-19 vaccine protection, but vaccine boosters can boost immunity (77) and improve the efficacy by 12-35 fold (78). However, most of the neutralizing monoclonal antibodies which are approved by the FDA for the treatment of SARS‐CoV‐2 variants, are ineffective against the Omicron variant except for Sotrovimab, and recent studies revealed that the Omicron BA.2 is also resistant to Sotrovimab (79). New Omicron variants BA.4 and BA.5 are reported to be more transmissible and can escape from BA.1 elicited immunity which is moderated by vaccination (80), and most monoclonal antibodies (81). Thus, the development of bivalent or multivalent vaccines that are designed to target new Omicron subvariants (BA.4 and BA.5) is urgently resolved (82).

### SARS-CoV-2 humoral immunity and cellular immunity

Currently, decreased immunity over time and reduced efficiency against variants appear to be major problems with many COVID-19 vaccines. As key immunological markers, NA can signal the elicitation of defense responses for the prevention and control of viral infections and disease onset. There is a correlation between the level of NA responses to SARS-CoV-2 and the protection level of the vaccine (66, 83), and increasing transmissibility and the reduced protective effects of NA have been proven to contribute to the rapid spread of Delta and Omicron variants (84–86). Among the current common vaccines, ZF2001 shows neutralizing activity against wild-type virus and Delta variant (87), however, it is nearly impossible to neutralize the Omicron variant (88). The neutralization efficiency with two doses of BNT162b2 vaccine against the Omicron variant is lower than wild-type virus (89, 90). Study also reported that after two doses of inactivated BBIBP-CorV, the neutralizing activity against wild-type virus is 80%, and only 10% of serum samples show successful neutralization against Omicron variants (91). Booster mRNA vaccines (BNT162b and mRNA-1273) increase NA responses, especially against Omicron variants, compared to two doses of mRNA vaccines. Neutralization against the Omicron variant is detected in all serum samples after the third dose of the mRNA-1273 vaccine, but not in some serum samples after the two doses of the mRNA-1273 vaccine (92, 93). Nemet et al. (90)reported that a third dose of the BNT162b2 vaccine was 100 times more effective in neutralizing the Omicron variant than the two-dose vaccination. The neutralization titers against wild-type virus and Omicron variant increase after the third inactivated vaccine (91). The neutralization titer of the BBIBP-CorV/ZF2001 heterologous booster group is higher relative to the homologous booster group (94). Both homologous and heterologous inactivated vaccine booster increase NA and improve the protection against the Omicron variant (91). It can be seen that it becomes necessary to continue vaccination after the vaccine booster and to strengthen the heterologous booster to improve the NA response against SARS-CoV-2 variants, especially emerging variants such as the Omicron variant.

In the process of severe COVID-19, the inflammatory response increased, and the number of T cells such as CD4^+^ and CD8^+^ T cells decreased significantly (57, 95–97), but activation of T cells increase (98, 99). CD4^+^ T cells can not only optimize the cytotoxic function and memory generation of CD8^+^ T cells but also play an important role in the production of highly effective neutralizing antibodies and the development of memory B cells (100, 101). Studies have demonstrated that the faster the response of antibodies and T cells to SARS-CoV-2, the better the improvement of infection outcomes (102, 103). Coordinated T-cell and antibody responses can reduce the severity of SARS-CoV-2 infection (104), and memory T-cell responses play a major role in immune protection against SARS-CoV-2 infection (105–107). In the case of poor antibody responses, CD8 T cell responses assume the main task of preventing severe COVID-19 (103, 108–110), while CD4 T cells are essential for protective antibody responses and promote the maturation and proliferation of CD8 T cells (111), which may contribute to recovery from COVID-19 infection when humoral immunity is deficient (112). Strong T-cell responses are associated with milder COVID-19, and they also contribute to the protective response produced by vaccination (103, 104). Studying SARS-CoV-2-specific T-cell responses could contribute to speeding up the vaccine testing process and facilitate vaccine development in the context of the COVID-19 pandemic (113). However, many unknowns remain, such as whether rapid induction of adaptive immune responses to SARS-CoV-2 and less severe COVID-19 manifestations are driven by pre-existing T cells (114), and whether a role for vaccine-elicited memory CD4^+^ T cell and CD8^+^ T cell responses in directly mediating protection from SARS-CoV-2 infection is unclear (115).

### Vaccines

On March 1, 2022, among five vaccines approved by EMA to prevent COVID-19, two mRNA vaccines were included: BNT162b2 and mRNA-1273 (116). BNT162b2 is a 30 μg dose of the SARS-CoV-2 spike protein encoded by mRNA, developed by BioNTech and Pfizer. Studies have shown that BNT162b2 has certain effectiveness in different populations (117–119) and different variants (118, 120–123). For immunocompromised patients, a recent clinical trial indicated that BioNTech was effective in 72.2% of immunocompromised individuals compared to 100% of healthy controls (124). Although BNT162b2 can elicit a significant immune response in most patients with autoimmune disease, the patient’s treatment may hinder the antibody response to vaccination (125–127). BNT162b2 is immunogenic in patients with retained immunity, although it is slightly less effective in HIV patients (128). However, serious adverse reactions including lymphadenopathy, severe chest pain, hypertension, allergy, paroxysmal ventricular arrhythmia, etc. have caused scholars’ attention (129).

### Cytokine storm

The cytokine storm of COVID-19 is an uncontrolled immune response of the host to SARS-CoV-2 infection, characterized by a sharp increase in proinflammatory cytokines (130). These cytokines can trigger immune cells to release a large number of free radicals, which can lead to acute respiratory distress syndrome (ARDS), accumulate into systemic inflammation, and eventually lead to multiple system organ failure (131). These cytokines mainly include interleukin-1 (IL-1), 2, 6, 7, 8, 10, 12, 17, 18; tumor necrosis factor-α (TNF-α); IFN-γ; granulocyte colony-stimulating factor; granulocyte-macrophage colony-stimulating factor (GM-CSF); and monocyte chemoattractant protein-1 (132). Studies have shown that these cytokines are associated with a variety of signal pathways: such as IL-2 and JAK-STAT5 signaling pathway (132), IL-7/IL-7R and JAK/STAT pathway (133), IL-10 and JAK/STAT3 pathway (134), and IL-6与JAK/STAT3 pathway (96, 135), whose activation can trigger inflammation, leading to the recruitment of lung cells, endothelial cells, macrophages, monocytes, lymphocytes, natural killer cells and dendritic cells into a cytokine storm (136). In addition, the IL-12 signaling pathway is involved in increasing the activation of immune cells, promoting the proliferation of Th1 and Th17 cells, and inducing the expression of IFN- γ (137). There are interactions between these cytokines, such as IFN- γ, IL-4 and IL-10 may inhibit the production of GM-CSF (138), and GM-CSF can regulate other cytokine signals (such as IL-1, IL-6, IL-2, IL-8, IL-10, and TNF-α) to influence the inflammatory response (139). Currently, monoclonal antibodies against a variety of cytokines and a variety of related signaling pathway modulatory agents have shown potential to control the SARS-CoV-2 cytokine storm, such as tocilizumab for IL-6 (140, 141), emapalumab for IFN-γ (142), canakinumab for IL-1β and anakinra for IL-1R (143), JAK2/STAT3 inhibitors (131, 144), NF-κB pathway regulators (145), MAPK/NF-κB pathway regulators (146), the nucleotide-binding and oligomerization domain-like receptor family pyrin domain-containing 3 inflammasome signaling inhibitors (147, 148). In addition, immunoglobulin (149), nanotechnology-based corticosteroid drugs (150), and traditional Chinese medicine (151–153) are also potential drugs to alleviate cytokine storms.

### Long COVID

Up to 70% of COVID-19 survivors infected with SARS-CoV-2 may develop long-term medical complications (154–157). Such symptoms are often referred to as “long COVID” (158, 159). There was no consistent definition of long COVID, so WHO provided a clinical definition: “Post-COVID-19 condition occurs in individuals with a history of probable or confirmed SARS-CoV-2 infection, usually 3 months from the onset of COVID-19 with symptoms that last for at least 2 months and cannot be explained by an alternative diagnosis.” (160). Long COVID patients often have respiratory dysfunction (161), cardiovascular complications (162, 163), neurological symptoms (164, 165), and gastrointestinal symptoms (166), etc. Chronic damage to these multiple system organs has a significant negative impact on the quality of life of long COVID patients (167). The pathophysiology is unknown. Residual viral particles and viral shedding have been proposed as probable causes of long COVID (168). Long COVID risk among fully SARS-CoV-2-vaccinated individuals is markedly reduced in comparison with the unvaccinated (169–171). However, the strength of the current evidence is limited, and further studies are required to determine the impact of vaccination on long COVID symptoms. A comprehensive multidisciplinary approach, including physical and mental rehabilitation services, to manage long COVID patients is extremely necessary. In addition, antiviral therapy shows great promise in reducing the incidence rate of long COVID (168).

Although no specific therapeutic regimens have been approved for COVID-19 to date, studies of interferon-based (172, 173) and antibody-based immunotherapies (174, 175), as well as polyclonal antibodies against SARS-CoV-2 (176, 177), support their clinical use as treatment options for COVID-19. In addition, the immunological efficacy of many traditional Chinese medicines has been confirmed. For example, Lianhua Qingwen Capsule can inhibit the cytopathic effect of SARS-CoV-2 *in vitro*, reduce the viral load in the cytoplasm and membrane (178), and inhibit the replication of SARS-CoV-2 (179). Shufeng Jiedu capsule can increase the expression of the A2A adenosine receptor, inhibit NF-κB phosphorylation, reduce inflammation and inhibit apoptosis and ultimately improve acute lung injury in COVID-19 (180). Studies have demonstrated the immunological efficacy of components of Chinese medicine in treating COVID-19: for example, components in Ephedra sinica can alleviate pneumonia symptoms by reducing the production of IL-6, IL-8, TNF-α, and MMP-9 (179), and quinoline-2-carboxylic acids in Ephedra sinica can disrupt the interaction between ACE2 and SARS-CoV-2 RBD (181). Extracts of Scutellaria baicalensis are able to inhibit the activity of SARS-CoV-2 3C-like protease and suppress SARS-CoV-2 replication *in vitro (* 182). Sanghuangporus sanghuang can significantly decrease the expression of ACE2 and transmembrane protease serine 2 and reduce their expression in mouse liver and kidney tissues (183). In addition, many natural products (gallinamide A, cordycepin, telocinobufagin, plitidepsin, and tylophorine) are able to target the SARS-CoV-2 main protease (Mpro), RNA-dependent RNA polymerase, papain-like protease, and spike glycoprotein, manifesting potential functions in the treatment of COVID-19 (184). However, the efficacy and safety of these Chinese medicine preparations and natural products need to be further evaluated. Notably, novel biomaterials such as nanomaterials have also shown positive effects on enhancing the drug efficacy of COVID-19. Thanks to the ability of lipid nanoparticles to protect encapsulated mRNA (185), lipid nanoparticle-based mRNA vaccines have received ‘emergency use authorization’ (by FDA) and ‘conditional approval’ by EMA. In addition, since SARS-CoV-2 infection occurs mainly in the epithelial cells of the respiratory tract, lipids/polymers can be configured as inhalable aerosols for the delivery of drugs such as curcumin, hesperidin, favipiravir, and therapeutic siRNAs targeting COVID-19 and its resulting cytokine storm, acute lung injury, and ARDS (186–190), which both avoid the side effects caused by intravenous systemic administration and first-pass metabolism (189) and overcome, to some extent, the barriers that exist in the pulmonary system (191). However, how to avoid the side effects caused by local deposition of inhaled drugs in the oral cavity and pharynx deserves in-depth study.

Combining the results of the dual-map overlays of Part A and B, it can be seen that, the published articles related to COVID-19 are mainly focused on journals in the field of molecular, biology, immunology, and medicine, medical, and clinical, whereas most the cited articles are published in journals in the field of molecular, biology, genetics, and health, nursing, medicine (**Figure 7**). It is gratifying that these COVID-19 related studies have reflected the effective penetration of multiple disciplines, not just immunology, which is also the current development trend of various disciplines.

### Inspiration and prospect

Conducting comparative studies on the epidemiology, genetic alterations and clinical manifestations of SARS-CoV-2 variants and their sublines will help to control the spread, diagnosis, detection, prevention, and treatment of the variants. In addition, understanding the roles of multiple viral enzymes such as RNA-dependent RNA polymerase, replicase, and transcriptase can help provide insight into the RNA replication process of SARS-CoV-2 and the development of modulators targeting these enzymes (192). Currently, the most critical measures to prevent and control the transmission of multiple variants such as Delta and Omicron remain to improve vaccination coverage, and there is a need to test the efficacy of vaccine cocktails against emerging variants, including Omicron (193), however, in-depth studies are needed to achieve long-term immunity. Besides, the COVID-19 vaccine or drug delivery system based on new materials such as nanoparticles will be an important research direction (194). The very important role of spike protein and RBD in protective humoral immunity induced by T-cell responses and neutralizing antibodies during SARS-CoV-2 infection provides an important direction for the development of new vaccines and drugs. Impaired function or overactivation of T cells may lead to serious complications in COVID-19 patients (195, 196), and therefore more data on T cell responses in large clinical trials of vaccines involving different populations are needed to better assess the role of T cells in SARS-CoV-2 infection. In addition, mechanisms by which SARS-CoV-2 evades recognition by T cells, including sequence mutations, and active alterations of antigen processing and presentation, need to be considered. Cytokine storm is strongly associated with fatal outcomes in patients with severe COVID-19 (197). Targeting these inflammatory cytokines as well as the application of immunomodulatory agents could benefit COVID-19 patients and enhance the effectiveness of antiviral therapy. However, due to the complexity of the inflammatory network, targeting one inflammatory factor pathway may stimulate downstream compensatory immune responses, and therefore the risks and benefits of these agents need to be balanced. SARS-CoV-2-mediated cytotoxicity, excessive inflammation, cytokine storm, and reactive physiological changes in the corresponding organs may combine to cause specific symptoms of long COVID. Current research on the pathophysiological mechanisms of long COVID has focused on the acute COVID-19 phase and subsequent organ dysfunction. However, as symptoms change over time, there is a need for pathophysiological studies at different time points. Vaccines, especially mRNA vaccines, have shown a preliminary potential to reduce the symptoms and prevent the progression of long COVID, but the duration of protection and its effectiveness against emerging variants still need further study.

### Limitations

Our study had several limitations. First, as an important bibliometric research database, the WOS database might still omit some important literature. Second, we used multiple software to analyze the contents of the 10 immunology journals with the highest IF and the 10 immunology journals with the most publications on COVID-19 in terms of countries, institutions, authors, keywords and references. However, we have to admit that the heterogeneity of this study existed because other immunology journals not included in our research might have published some important papers. However, we believe that the literature included in this study may, to a large extent, enable scholars to quickly understand what important immunological information, research hotspots, and development trends the articles on COVID-19 published in major immunology journals have transmitted.

## Conclusion

Since the COVID-19 global pandemic, the USA, China, England, and Italy have made the most significant contributions to the studies on COVID-19 published in major immunology journals. Although the number of articles was not the largest, UCL, NIAID, Harvard Medical School, University California San Diego, and University of Pennsylvania played a central role in international cooperation in the immunology research field of COVID-19. Yuen Kwok Yung from the University of Hong Kong was the most important author in terms of the number of publications and citations, and the H-index. As a group author, WHO was the most cited. From the perspective of the number of publications and citations, and H-index, *CLINICAL INFECTIOUS DISEASES* and *FRONTIERS IN IMMUNOLOGY* were the most important immunology journals in the field of COVID-19-related research. Currently, the most essential immunology journals focused on the following topics: “Delta/Omicron variants”, “cytokine storm”, “neutralization/neutralizing antibody”, “T cell”, “BNT162b2”, “mRNA vaccine”, “vaccine effectiveness/safety”, and “long COVID”.

## Data availability statement

The original contributions presented in the study are included in the article/**Supplementary Material**. Further inquiries can be directed to the corresponding authors.

## Author contributions

JZha, RY, and QW contributed to the study conception design. JZha, JZhu, CH, and ZZ contributed to the acquisition, analysis, and interpretation of data. Manuscript draft and revision: all authors. All authors read and approved the final manuscript.

## Funding

The study was supported by the National Natural Science Foundation of China (Grant Number: 81960436), Project of the Jiangxi Provincial Department of Science and Technology (Grant Number: 20202BBGL73037), and Double Thousand Talents Project of Jiangxi Province (Grant Number: jxsq2019201100).

## Acknowledgments

We thank the World Health Organization for their great contribution to the global fight against COVID-19.

## Conflict of interest

The authors declare that the research was conducted in the absence of any commercial or financial relationships that could be construed as a potential conflict of interest.

## Publisher’s note

All claims expressed in this article are solely those of the authors and do not necessarily represent those of their affiliated organizations, or those of the publisher, the editors and the reviewers. Any product that may be evaluated in this article, or claim that may be made by its manufacturer, is not guaranteed or endorsed by the publisher.

## Supplementary material

Supplementary Table 1 The top 25 institutions with the most publications.

Supplementary Table 2 Top 10 highly co-cited references.

## References

[1] Tu B Gao Y An X Wang H Huang Y. Localized delivery of nanomedicine and antibodies for combating covid-19. Acta Pharm Sin B (2022). doi: 10.1016/j.apsb.2022.09.011 PMC9502448 36168329

[2] Ankerhold J Giese S Kolb P Maul-Pavicic A Voll RE Göppert N. Circulating multimeric immune complexes contribute to immunopathology in covid-19. Nat Commun (2022) 13(1):5654. doi: 10.1038/s41467-022-32867-z 36163132 PMC9513013

[3] Kolb P Giese S Voll RE Hengel H Falcone V. Immune complexes as culprits of immunopathology in severe covid-19. Med Microbiol Immunol (2022), 1–7. doi: 10.1007/s00430-022-00743-8 35871171 PMC9308473

[4] Okuya K Hattori T Saito T Takadate Y Sasaki M Furuyama W. Multiple routes of antibody-dependent enhancement of sars-Cov-2 infection. Microbiol Spectr (2022) 10(2):e0155321. doi: 10.1128/spectrum.01553-21 35319248 PMC9045191

[5] Lim EHT van Amstel RBE de Boer VV van Vught LA de Bruin S Brouwer MC. Complement activation in covid-19 and targeted therapeutic options: A scoping review. Blood Rev (2022) 100995. doi: 10.1016/j.blre.2022.100995 PMC9338830 35934552

[6] Afzali B Noris M Lambrecht BN Kemper C. The state of complement in covid-19. Nat Rev Immunol (2022) 22(2):77–84. doi: 10.1038/s41577-021-00665-1 34912108 PMC8672651

[7] Boussier J Yatim N Marchal A Hadjadj J Charbit B El Sissy C. Severe covid-19 is associated with hyperactivation of the alternative complement pathway. J Allergy Clin Immunol (2022) 149(2):550–6.e2. doi: 10.1016/j.jaci.2021.11.004 34800432 PMC8595971

[8] Mastaglio S Ruggeri A Risitano AM Angelillo P Yancopoulou D Mastellos DC. The first case of covid-19 treated with the complement C3 inhibitor Amy-101. Clin Immunol (2020) 215:108450. doi: 10.1016/j.clim.2020.108450 32360516 PMC7189192

[9] Carvelli J Demaria O Vely F Batista L Chouaki Benmansour N Fares J. Association of covid-19 inflammation with activation of the C5a-C5ar1 axis. Nature (2020) 588(7836):146–50. doi: 10.1038/s41586-020-2600-6 PMC7116884 32726800

[10] McEneny-King AC Monteleone JPR Kazani SD Ortiz SR. Pharmacokinetic and pharmacodynamic evaluation of ravulizumab in adults with severe coronavirus disease 2019. Infect Dis Ther (2021) 10(2):1045–54. doi: 10.1007/s40121-021-00425-7 PMC8024938 33826106

[11] Ruggenenti P Di Marco F Cortinovis M Lorini L Sala S Novelli L. Eculizumab in patients with severe coronavirus disease 2019 (Covid-19) requiring continuous positive airway pressure ventilator support: Retrospective cohort study. PloS One (2021) 16(12):e0261113. doi: 10.1371/journal.pone.0261113 34928990 PMC8687582

[12] Kalil AC Proschan M. Complement C5a inhibition: A new form of covid-19 treatment for mechanically ventilated patients? Lancet Respir Med (2022). doi: 10.1016/s2213-2600(22)00365-4 PMC9467518 36108660

[13] Al-Kuraishy HM Al-Gareeb AI Jalal NA Kabrah SM Alexiou A Batiha GE. Sars-Cov-2 infection and C1-esterase inhibitor: Camouflage pattern and new perspective. Curr Protein Pept Sci (2022). doi: 10.2174/1389203723666220811121803 35959625

[14] De Leeuw E Van Damme KFA Declercq J Bosteels C Maes B Tavernier SJ. Efficacy and safety of the investigational complement C5 inhibitor zilucoplan in patients hospitalized with covid-19: An open-label randomized controlled trial. Respir Res (2022) 23(1):202. doi: 10.1186/s12931-022-02126-2 35945604 PMC9361275

[15] Mastellos DC Pires da Silva BGP Fonseca BAL Fonseca NP Auxiliadora-Martins M Mastaglio S. Complement C3 vs C5 inhibition in severe covid-19: Early clinical findings reveal differential biological efficacy. Clin Immunol (2020) 220:108598. doi: 10.1016/j.clim.2020.108598 32961333 PMC7501834

[16] He W Gao Y Zhou J Shi Y Xia D Shen HM. Friend or foe? implication of the autophagy-lysosome pathway in sars-Cov-2 infection and covid-19. Int J Biol Sci (2022) 18(12):4690–703. doi: 10.7150/ijbs.72544 PMC9305279 35874956

[17] Ou X Liu Y Lei X Li P Mi D Ren L. Characterization of spike glycoprotein of sars-Cov-2 on virus entry and its immune cross-reactivity with sars-cov. Nat Commun (2020) 11(1):1620. doi: 10.1038/s41467-020-15562-9 32221306 PMC7100515

[18] Wrapp D Wang N Corbett KS Goldsmith JA Hsieh CL Abiona O. Cryo-em structure of the 2019-ncov spike in the prefusion conformation. Science (2020) 367(6483):1260–3. doi: 10.1126/science.abb2507 PMC7164637 32075877

[19] Wang B Zhao J Liu S Feng J Luo Y He X. Ace2 decoy receptor generated by high-throughput saturation mutagenesis efficiently neutralizes sars-Cov-2 and its prevalent variants. Emerg Microbes Infect (2022) 11(1):1488–99. doi: 10.1080/22221751.2022.2079426 PMC9176695 35587428

[20] Ankerhold J Giese S Kolb P Maul-Pavicic A Göppert N Ciminski K. Circulating immune complexes drive immunopathology in covid-19. bioRxiv (2021) 13(1):5654. doi: 10.1101/2021.06.25.449893 PMC9513013 36163132

[21] Rosenberg ES Dorabawila V Easton D Bauer UE Kumar J Hoen R. Covid-19 vaccine effectiveness in new York state. N Engl J Med (2022) 386(2):116–27. doi: 10.1056/NEJMoa2116063 PMC8693697 34942067

[22] Thompson MG Stenehjem E Grannis S Ball SW Naleway AL Ong TC. Effectiveness of covid-19 vaccines in ambulatory and inpatient care settings. N Engl J Med (2021) 385(15):1355–71. doi: 10.1056/NEJMoa2110362 PMC8451184 34496194

[23] Patel R Kaki M Potluri VS Kahar P Khanna D. A comprehensive review of sars-Cov-2 vaccines: Pfizer, moderna & Johnson & Johnson. Hum Vaccin Immunother (2022) 18(1):2002083. doi: 10.1080/21645515.2021.2002083 35130825 PMC8862159

[24] Herman GA O'Brien MP Forleo-Neto E Sarkar N Isa F Hou P. Efficacy and safety of a single dose of casirivimab and imdevimab for the prevention of covid-19 over an 8-month period: A randomised, double-blind, placebo-controlled trial. Lancet Infect Dis (2022) 22(10):1444–54. doi: 10.1016/s1473-3099(22)00416-9 PMC9255947 35803290

[25] Montgomery H Hobbs FDR Padilla F Arbetter D Templeton A Seegobin S. Efficacy and safety of intramuscular administration of tixagevimab-cilgavimab for early outpatient treatment of covid-19 (Tackle): A phase 3, randomised, double-blind, placebo-controlled trial. Lancet Respir Med (2022) 10(10):985–96. doi: 10.1016/s2213-2600(22)00180-1 PMC9173721 35688164

[26] ACTIV-3–Therapeutics for Inpatients with COVID-19 (TICO) Study Group. Tixagevimab-cilgavimab for treatment of patients hospitalised with covid-19: A randomised, double-blind, phase 3 trial. Lancet Respir Med (2022) 10(10):972–84. doi: 10.1016/s2213-2600(22)00215-6 PMC9270059 35817072

[27] Maeda R Fujita J Konishi Y Kazuma Y Yamazaki H Anzai I. A panel of nanobodies recognizing conserved hidden clefts of all sars-Cov-2 spike variants including omicron. Commun Biol (2022) 5(1):669. doi: 10.1038/s42003-022-03630-3 35794202 PMC9257560

[28] Weinstein JB Bates TA Leier HC McBride SK Barklis E Tafesse FG. A potent alpaca-derived nanobody that neutralizes sars-Cov-2 variants. iScience (2022) 25(3):103960. doi: 10.1016/j.isci.2022.103960 35224467 PMC8863326

[29] Yu B Li S Tabata T Wang N Cao L Kumar GR. Accelerating perx reaction enables covalent nanobodies for potent neutralization of sars-Cov-2 and variants. Chem (2022) 8(10):2766–83. doi: 10.1101/2022.03.11.483867 PMC9288967 35874165

[30] Hong J Kwon HJ Cachau R Chen CZ Butay KJ Duan Z. Dromedary Camel Nanobodies Broadly Neutralize Sars-Cov-2 Variants. Proc Natl Acad Sci USA (2022) 119(18):e2201433119. doi: 10.1073/pnas.2201433119 35476528 PMC9170159

[31] Güttler T Aksu M Dickmanns A Stegmann KM Gregor K Rees R. Neutralization of sars-Cov-2 by highly potent, hyperthermostable, and mutation-tolerant nanobodies. EMBO J (2021) 40(19):e107985. doi: 10.15252/embj.2021107985 34302370 PMC8420576

[32] Zhang Q Li S Liu J Chen J. Global trends in nursing-related research on covid-19: A bibliometric analysis. Front Public Health (2022) 10:933555. doi: 10.3389/fpubh.2022.933555 35923953 PMC9339968

[33] Li C Li Z Guo J Yang Y Liu C Wang M. The hotspots and trends of coronavirus disease 2019 (Covid-19) and physical therapy: A bibliometric and visual analysis. J Phys Ther Sci (2021) 33(12):903–7. doi: 10.1589/jpts.33.903 PMC8636920 34873371

[34] Xia D Yao R Wang S Chen G Wang Y. Mapping trends and hotspots regarding clinical research on covid-19: A bibliometric analysis of global research. Front Public Health (2021) 9:713487. doi: 10.3389/fpubh.2021.713487 34497794 PMC8419357

[35] Sanchez-Tena MA Martinez-Perez C Villa-Collar C Alvarez-Peregrina C. Impact of covid-19 at the ocular level: A citation network study. J Clin Med (2021) 10(7):1340. doi: 10.3390/jcm10071340 33804977 PMC8036864

[36] Cheng K Guo Q Yang W Wang Y Sun Z Wu H. Mapping knowledge landscapes and emerging trends of the links between bone metabolism and diabetes mellitus: A bibliometric analysis from 2000 to 2021. Front Public Health (2022) 10:918483. doi: 10.3389/fpubh.2022.918483 35719662 PMC9204186

[37] Chen C Leydesdorff L. Patterns of connections and movements in dual-map overlays: A new method of publication portfolio analysis. J Assoc Inf Sci Technol (2014) 65(2):334–51. doi: 10.1002/asi.22968

[38] Dai Z Xu S Wu X Hu R Li H He H. Knowledge mapping of multicriteria decision analysis in healthcare: A bibliometric analysis. Front Public Health (2022) 10:895552. doi: 10.3389/fpubh.2022.895552 35757629 PMC9218106

[39] Zhao JJ Yuan Q Wang HY Liu W Liao XJ Su YY. Antibody responses to sars-Cov-2 in patients with novel coronavirus disease 2019. Clin Infect Dis (2020) 71(16):2027–34. doi: 10.1093/cid/ciaa344 PMC7184337 32221519

[40] Qin C Zhou LQ Hu ZW Zhang SQ Yang S Tao Y. Dysregulation of immune response in patients with coronavirus 2019 (Covid-19) in wuhan, China. Clin Infect Dis (2020) 71(15):762–8. doi: 10.1093/cid/ciaa248 PMC7108125 32161940

[41] Chan JFW Kok KH Zhu Z Chu H To KKW Yuan SF. Genomic characterization of the 2019 novel human-pathogenic coronavirus isolated from a patient with atypical pneumonia after visiting wuhan. Emerging Microbes Infections (2020) 9(1):221–36. doi: 10.1080/22221751.2020.1719902 PMC7067204 31987001

[42] Yao XT Ye F Zhang M Cui C Huang BY Niu PH. In vitro antiviral activity and projection of optimized dosing design of hydroxychloroquine for the treatment of severe acute respiratory syndrome coronavirus 2 (Sars-Cov-2). Clin Infect Dis (2020) 71(15):732–9. doi: 10.1093/cid/ciaa237 PMC7108130 32150618

[43] Wang CY Pan RY Wan XY Tan YL Xu LK McIntyre RS. A longitudinal study on the mental health of general population during the covid-19 epidemic in China. Brain Behav And Immun (2020) 87:40–8. doi: 10.1016/j.bbi.2020.04.028 PMC7153528 32298802

[44] To KKW Tsang OTY Yip CCY Chan KH Wu TC Chan JMC. Consistent detection of 2019 novel coronavirus in saliva. Clin Infect Dis (2020) 71(15):841–3. doi: 10.1093/cid/ciaa149 PMC7108139 32047895

[45] Zhang W Du RH Li B Zheng XS Yang XL Hu B. Molecular and serological investigation of 2019-ncov infected patients: Implication of multiple shedding routes. Emerging Microbes Infections (2020) 9(1):386–89. doi: 10.1080/22221751.2020.1729071 PMC7048229 32065057

[46] Wu YS Xu XL Chen ZJ Duan JH Hashimoto K Yang L. Nervous system involvement after infection with covid-19 and other coronaviruses. Brain Behav And Immun (2020) 87:18–22. doi: 10.1016/j.bbi.2020.03.031 PMC7146689 32240762

[47] Guo L Ren LL Yang SY Xiao M Chang D Yang F. Profiling early humoral response to diagnose novel coronavirus disease (Covid-19). Clin Infect Dis (2020) 71(15):778–85. doi: 10.1093/cid/ciaa310 PMC7184472 32198501

[48] Tai WB He L Zhang XJ Pu J Voronin D Jiang SB. Characterization of the receptor-binding domain (Rbd) of 2019 novel coronavirus: Implication for development of rbd protein as a viral attachment inhibitor and vaccine. Cell Mol Immunol (2020) 17(6):613–20. doi: 10.1038/s41423-020-0400-4 PMC7091888 32203189

[49] Consortium WHOST. Repurposed antiviral drugs for covid-19-Interim who solidarity trial results. New Engl J Of Med (2021) 384(6):497–511. doi: 10.1056/NEJMoa2023184 33264556 PMC7727327

[50] Sterne JAC Murthy S Diaz JV Slutsky AS Villar J Angus DC. Association between administration of systemic corticosteroids and mortality among critically ill patients with covid-19 a meta-analysis. Jama-Journal Of Am Med Assoc (2020) 324(13):1330–41. doi: 10.1001/jama.2020.17023 PMC7489434 32876694

[51] Coates A Warren KT Henderson C McPherson M Obubah O Graaff P. The world health organization's frontline support to countries during the covid-19 pandemic in 2020. Front Public Health (2022) 10:850260. doi: 10.3389/fpubh.2022.850260 35372256 PMC8971552

[52] Singh JA Kochhar S Wolff J Atuire C Bhan A Emanuel E. Who guidance on covid-19 vaccine trial designs in the context of authorized covid-19 vaccines and expanding global access: Ethical considerations. Vaccine (2022) 40(14):2140–9. doi: 10.1016/j.vaccine.2022.02.038 PMC8882397 35248422

[53] Knezevic I Mattiuzzo G Page M Minor P Griffiths E Nuebling M. Who international standard for evaluation of the antibody response to covid-19 vaccines: Call for urgent action by the scientific community. Lancet Microbe (2022) 3(3):e235–e40. doi: 10.1016/s2666-5247(21)00266-4 PMC8547804 34723229

[54] Badu K Oyebola K Zahouli JZB Fagbamigbe AF de Souza DK Dukhi N. Sars-Cov-2 viral shedding and transmission dynamics: Implications of who covid-19 discharge guidelines. Front Med (Lausanne) (2021) 8:648660. doi: 10.3389/fmed.2021.648660 34239886 PMC8259580

[55] Who Guidelines Approved by the Guidelines Review Committee. Drugs to prevent covid-19: A who living guideline Vol. 2021. . Geneva: World Health Organization© (2021). 35917395

[56] Ngoy N Oyugi B Ouma PO Conteh IN Woldetsadik SF Nanyunja M. Coordination mechanisms for covid-19 in the who regional office for Africa. BMC Health Serv Res (2022) 22(1):711. doi: 10.1186/s12913-022-08035-w 35643550 PMC9142827

[57] Huang CL Wang YM Li XW Ren LL Zhao JP Hu Y. Clinical features of patients infected with 2019 novel coronavirus in wuhan, China. Lancet (2020) 395(10223):497–506. doi: 10.1016/S0140-6736(20)30183-5 31986264 PMC7159299

[58] Zhou F Yu T Du RH Fan GH Liu Y Liu ZB. Clinical course and risk factors for mortality of adult inpatients with covid-19 in wuhan, China: A retrospective cohort study. LANCET (2020) 395(10229):1054–62. doi: 10.1016/S0140-6736(20)30566-3 PMC7270627 32171076

[59] Zhu N Zhang DY Wang WL Li XW Yang B Song JD. A novel coronavirus from patients with pneumonia in China, 2019. New Engl J Of Med (2020) 382(8):727–33. doi: 10.1056/NEJMoa2001017 PMC7092803 31978945

[60] Guan W Ni Z Hu Y Liang W Ou C He J. Clinical characteristics of coronavirus disease 2019 in China. New Engl J Of Med (2020) 382(18):1708–20. doi: 10.1056/NEJMoa2002032 PMC7092819 32109013

[61] Sallam M. Covid-19 vaccine hesitancy worldwide: A concise systematic review of vaccine acceptance rates. VACCINES (2021) 9(2):160. doi: 10.3390/vaccines9020160 33669441 PMC7920465

[62] Diao B Wang CH Tan YJ Chen XW Liu Y Ning LF. Reduction and functional exhaustion of T cells in patients with coronavirus disease 2019 (Covid-19). Front In Immunol (2020) 11:827. doi: 10.3389/fimmu.2020.00827 32425950 PMC7205903

[63] Reiter PL Pennell ML Katz ML. Acceptability of a covid-19 vaccine among adults in the united states: How many people would get vaccinated? Vaccine (2020) 38(42):6500–7. doi: 10.1016/j.vaccine.2020.08.043 PMC7440153 32863069

[64] Detoc M Bruel S Frappe P Tardy B Botelho-Nevers E Gagneux-Brunon A. Intention to participate in a covid-19 vaccine clinical trial and to get vaccinated against covid-19 in France during the pandemic. Vaccine (2020) 38(45):7002–6. doi: 10.1016/j.vaccine.2020.09.041 PMC7498238 32988688

[65] Ruiz JB Bell RA. Predictors of intention to vaccinate against covid-19: Results of a nationwide survey. Vaccine (2021) 39(7):1080–6. doi: 10.1016/j.vaccine.2021.01.010 PMC7794597 33461833

[66] Earle KA Ambrosino DM Fiore-Gartland A Goldblatt D Gilbert PB Siber GR. Evidence for antibody as a protective correlate for covid-19 vaccines. Vaccine (2021) 39(32):4423–8. doi: 10.1016/j.vaccine.2021.05.063 PMC8142841 34210573

[67] Wang KL Wong ELY Ho KF Cheung AWL Chan EYY Yeoh EK. Intention of nurses to accept coronavirus disease 2019 vaccination and change of intention to accept seasonal influenza vaccination during the coronavirus disease 2019 pandemic: A cross-sectional survey. VACCINE (2020) 38(45):7049–56. doi: 10.1016/j.vaccine.2020.09.021 PMC7834255 32980199

[68] Wong MCS Wong ELY Huang JJ Cheung AWL Law K Chong MKC. Acceptance of the covid-19 vaccine based on the health belief model: A population-based survey in Hong Kong. VACCINE (2021) 39(7):1148–56. doi: 10.1016/j.vaccine.2020.12.083 PMC7832076 33461834

[69] Wang X Li D Huang X Luo Q Li X Zhang X. A bibliometric analysis and visualization of photothermal therapy on cancer. Transl Cancer Res (2021) 10(3):1204–15. doi: 10.21037/tcr-20-2961 PMC8797757 35116448

[70] Liu T Yang L Mao H Ma F Wang Y Zhan Y. Knowledge domain and emerging trends in podocyte injury research from 1994 to 2021: A bibliometric and visualized analysis. Front Pharmacol (2021) 12:772386. doi: 10.3389/fphar.2021.772386 34925030 PMC8678497

[71] Liu C Ginn HM Dejnirattisai W Supasa P Wang B Tuekprakhon A. Reduced neutralization of sars-Cov-2 B.1.617 by vaccine and convalescent serum. Cell (2021) 184(16):4220–36.e13. doi: 10.1016/j.cell.2021.06.020 34242578 PMC8218332

[72] Xiang B Zhang Y Ling Q Xie Z Li N Wu D. Characteristics and management of sars-Cov-2 delta variant-induced covid-19 infections from may to October 2021 in China: Post-vaccination infection cases. Am J Transl Res (2022) 14(6):3603–9. PMC9274556 35836857

[73] Martinez DR Schäfer A Leist SR de la Cruz G West A Atochina-Vasserman EN. Chimeric spike mrna vaccines protect against sarbecovirus challenge in mice. Science (2021) 373(6558):991–8. doi: 10.1126/science.abi4506 PMC8899822 34214046

[74] Jawalagatti V Kirthika P Hewawaduge C Yang MS Park JY Oh B. Bacteria-enabled oral delivery of a replicon-based mrna vaccine candidate protects against ancestral and delta variant sars-Cov-2. Mol Ther (2022) 30(5):1926–40. doi: 10.1016/j.ymthe.2022.01.042 PMC8810265 35123065

[75] World Health Organization. Classification of omicron (B. 1.1. 529): Sars-Cov-2 variant of concern. Available at: https://www.who.int/news/item/26-11-2021-classification-of-omicron-(b.1.1.529)-sars-cov-2-variant-of-concern (Accessed November 26, 2021)

[76] Kumar S Karuppanan K Subramaniam G. Omicron (Ba.1) and Sub-variants (Ba.1.1, Ba.2, and Ba.3) of sars-Cov-2 spike infectivity and pathogenicity: A comparative sequence and structural-based computational assessment. J Med Virol (2022) 94(10):4780–91. doi: 10.1002/jmv.27927 PMC9347785 35680610

[77] Setiabudi D Sribudiani Y Hermawan K Andriyoko B Nataprawira HM. The omicron variant of concern: The genomics, diagnostics, and clinical characteristics in children. Front Pediatr (2022) 10:898463. doi: 10.3389/fped.2022.898463 35983081 PMC9378986

[78] Shrestha LB Foster C Rawlinson W Tedla N Bull RA. Evolution of the sars-Cov-2 omicron variants Ba.1 to Ba.5: Implications for immune escape and transmission. Rev Med Virol (2022) 32(5):e2381. doi: 10.1002/rmv.2381 35856385 PMC9349777

[79] Iketani S Liu L Guo Y Liu L Chan JF Huang Y. Antibody evasion properties of sars-Cov-2 omicron sublineages. Nature (2022) 604(7906):553–6. doi: 10.1038/s41586-022-04594-4 PMC9021018 35240676

[80] Khan K Karim F Ganga Y Bernstein M Jule Z Reedoy K. Omicron Ba.4/Ba.5 escape neutralizing immunity elicited by Ba.1 infection. Nat Commun (2022) 13(1):4686. doi: 10.1038/s41467-022-32396-9 35948557 PMC9364294

[81] Cao Y Yisimayi A Jian F Song W Xiao T Wang L. Ba.2.12.1, Ba.4 and Ba.5 escape antibodies elicited by omicron infection. Nature (2022) 608(7923):593–602. doi: 10.1038/s41586-022-04980-y 35714668 PMC9385493

[82] Marcotte H Hammarström L Pan-Hammarström Q. Limited cross-variant neutralization after primary omicron infection: Consideration for a variant-containing booster. Signal Transduct Target Ther (2022) 7(1):294. doi: 10.1038/s41392-022-01146-0 35995763 PMC9395412

[83] Khoury DS Cromer D Reynaldi A Schlub TE Wheatley AK Juno JA. Neutralizing antibody levels are highly predictive of immune protection from symptomatic sars-Cov-2 infection. Nat Med (2021) 27(7):1205–11. doi: 10.1038/s41591-021-01377-8 34002089

[84] Planas D Saunders N Maes P Guivel-Benhassine F Planchais C Buchrieser J. Considerable escape of sars-Cov-2 omicron to antibody neutralization. Nature (2022) 602(7898):671–5. doi: 10.1038/s41586-021-04389-z 35016199

[85] Zou J Xie X Liu M Shi PY Ren P. Neutralization titers in vaccinated patients with sars-Cov-2 delta breakthrough infections. mBio (2022) 13(4):e0199622. doi: 10.1128/mbio.01996-22 35924850 PMC9426493

[86] Guo Y Han J Zhang Y He J Yu W Zhang X. Sars-Cov-2 omicron variant: Epidemiological features, biological characteristics, and clinical significance. Front Immunol (2022) 13:877101. doi: 10.3389/fimmu.2022.877101 35572518 PMC9099228

[87] Zhao X Zheng A Li D Zhang R Sun H Wang Q. Neutralisation of Zf2001-elicited antisera to sars-Cov-2 variants. Lancet Microbe (2021) 2(10):e494. doi: 10.1016/s2666-5247(21)00217-2 34458880 PMC8378832

[88] Hu J Peng P Cao X Wu K Chen J Wang K. Increased immune escape of the new sars-Cov-2 variant of concern omicron. Cell Mol Immunol (2022) 19(2):293–5. doi: 10.1038/s41423-021-00836-z PMC8749347 35017716

[89] Collie S Champion J Moultrie H Bekker LG Gray G. Effectiveness of Bnt162b2 vaccine against omicron variant in south Africa. N Engl J Med (2022) 386(5):494–6. doi: 10.1056/NEJMc2119270 PMC8757569 34965358

[90] Nemet I Kliker L Lustig Y Zuckerman N Erster O Cohen C. Third Bnt162b2 vaccination neutralization of sars-Cov-2 omicron infection. N Engl J Med (2022) 386(5):492–4. doi: 10.1056/NEJMc2119358 PMC8823651 34965337

[91] Wang X Zhao X Song J Wu J Zhu Y Li M. Homologous or heterologous booster of inactivated vaccine reduces sars-Cov-2 omicron variant escape from neutralizing antibodies. Emerg Microbes Infect (2022) 11(1):477–81. doi: 10.1080/22221751.2022.2030200 PMC8820826 35034583

[92] Garcia-Beltran WF St Denis KJ Hoelzemer A Lam EC Nitido AD Sheehan ML. Mrna-based covid-19 vaccine boosters induce neutralizing immunity against sars-Cov-2 omicron variant. Cell (2022) 185(3):457–66.e4. doi: 10.1016/j.cell.2021.12.033 34995482 PMC8733787

[93] Pajon R Doria-Rose NA Shen X Schmidt SD O'Dell S McDanal C. Sars-Cov-2 omicron variant neutralization after mrna-1273 booster vaccination. N Engl J Med (2022) 386(11):1088–91. doi: 10.1056/NEJMc2119912 PMC8809504 35081298

[94] Ai J Zhang H Zhang Y Lin K Zhang Y Wu J. Omicron variant showed lower neutralizing sensitivity than other sars-Cov-2 variants to immune sera elicited by vaccines after boost. Emerg Microbes Infect (2022) 11(1):337–43. doi: 10.1080/22221751.2021.2022440 PMC8788341 34935594

[95] Mathew D Giles JR Baxter AE Oldridge DA Greenplate AR Wu JE. Deep immune profiling of covid-19 patients reveals distinct immunotypes with therapeutic implications. Science (2020) 369(6508):eabc8511. doi: 10.1126/science.abc8511 32669297 PMC7402624

[96] Giamarellos-Bourboulis EJ Netea MG Rovina N Akinosoglou K Antoniadou A Antonakos N. Complex immune dysregulation in covid-19 patients with severe respiratory failure. Cell Host Microbe (2020) 27(6):992–1000.e3. doi: 10.1016/j.chom.2020.04.009 32320677 PMC7172841

[97] Zhou Z Ren L Zhang L Zhong J Xiao Y Jia Z. Heightened innate immune responses in the respiratory tract of covid-19 patients. Cell Host Microbe (2020) 27(6):883–90.e2. doi: 10.1016/j.chom.2020.04.017 32407669 PMC7196896

[98] Lucas C Wong P Klein J Castro TBR Silva J Sundaram M. Longitudinal analyses reveal immunological misfiring in severe covid-19. Nature (2020) 584(7821):463–9. doi: 10.1038/s41586-020-2588-y PMC7477538 32717743

[99] Kuri-Cervantes L Pampena MB Meng W Rosenfeld AM Ittner CAG Weisman AR. Comprehensive mapping of immune perturbations associated with severe covid-19. Sci Immunol (2020) 5(49):eabd7114. doi: 10.1126/sciimmunol.abd7114 32669287 PMC7402634

[100] Seder RA Ahmed R. Similarities and differences in Cd4+ and Cd8+ effector and memory T cell generation. Nat Immunol (2003) 4(9):835–42. doi: 10.1038/ni969 12942084

[101] Kurosaki T Kometani K Ise W. Memory b cells. Nat Rev Immunol (2015) 15(3):149–59. doi: 10.1038/nri3802 25677494

[102] Lucas C Klein J Sundaram ME Liu F Wong P Silva J. Delayed production of neutralizing antibodies correlates with fatal covid-19. Nat Med (2021) 27(7):1178–86. doi: 10.1038/s41591-021-01355-0 PMC8785364 33953384

[103] Tan AT Linster M Tan CW Le Bert N Chia WN Kunasegaran K. Early induction of functional sars-Cov-2-Specific T cells associates with rapid viral clearance and mild disease in covid-19 patients. Cell Rep (2021) 34(6):108728. doi: 10.1016/j.celrep.2021.108728 33516277 PMC7826084

[104] Rydyznski Moderbacher C Ramirez SI Dan JM Grifoni A Hastie KM Weiskopf D. Antigen-specific adaptive immunity to sars-Cov-2 in acute covid-19 and associations with age and disease severity. Cell (2020) 183(4):996–1012.e19. doi: 10.1016/j.cell.2020.09.038 33010815 PMC7494270

[105] Sette A Crotty S. Adaptive immunity to sars-Cov-2 and covid-19. Cell (2021) 184(4):861–80. doi: 10.1016/j.cell.2021.01.007 PMC7803150 33497610

[106] Lipsitch M Grad YH Sette A Crotty S. Cross-reactive memory T cells and herd immunity to sars-Cov-2. Nat Rev Immunol (2020) 20(11):709–13. doi: 10.1038/s41577-020-00460-4 PMC7537578 33024281

[107] Bertoletti A Le Bert N Qui M Tan AT. Sars-Cov-2-Specific T cells in infection and vaccination. Cell Mol Immunol (2021) 18(10):2307–12. doi: 10.1038/s41423-021-00743-3 PMC8408362 34471260

[108] McMahan K Yu J Mercado NB Loos C Tostanoski LH Chandrashekar A. Correlates of protection against sars-Cov-2 in rhesus macaques. Nature (2021) 590(7847):630–4. doi: 10.1038/s41586-020-03041-6 PMC7906955 33276369

[109] Liao M Liu Y Yuan J Wen Y Xu G Zhao J. Single-cell landscape of bronchoalveolar immune cells in patients with covid-19. Nat Med (2020) 26(6):842–4. doi: 10.1038/s41591-020-0901-9 32398875

[110] Sekine T Perez-Potti A Rivera-Ballesteros O Strålin K Gorin JB Olsson A. Robust T cell immunity in convalescent individuals with asymptomatic or mild covid-19. Cell (2020) 183(1):158–68.e14. doi: 10.1016/j.cell.2020.08.017 32979941 PMC7427556

[111] Painter MM Mathew D Goel RR Apostolidis SA Pattekar A Kuthuru O. Rapid induction of antigen-specific Cd4(+) T cells is associated with coordinated humoral and cellular immunity to sars-Cov-2 mrna vaccination. Immunity (2021) 54(9):2133–42.e3. doi: 10.1016/j.immuni.2021.08.001 34453880 PMC8361141

[112] Bange EM Han NA Wileyto P Kim JY Gouma S Robinson J. Cd8(+) T cells contribute to survival in patients with covid-19 and hematologic cancer. Nat Med (2021) 27(7):1280–9. doi: 10.1038/s41591-021-01386-7 PMC8291091 34017137

[113] Wang Y Tian Q Ye L. The differentiation and maintenance of sars-Cov-2-Specific follicular helper T cells. Front Cell Infect Microbiol (2022) 12:953022. doi: 10.3389/fcimb.2022.953022 35909969 PMC9329515

[114] Niessl J Sekine T. Buggert m. T Cell Immun to Sars-Cov-2. Semin Immunol (2021) 55:101505. doi: 10.1016/j.smim.2021.101505 PMC8529278 34711489

[115] Kent SJ Khoury DS Reynaldi A Juno JA Wheatley AK Stadler E. Disentangling the relative importance of T cell responses in covid-19: Leading actors or supporting cast? Nat Rev Immunol (2022) 22(6):387–97. doi: 10.1038/s41577-022-00716-1 PMC9047577 35484322

[116] EMA. (Accessed August 7, 2022).

[117] Polack FP Thomas SJ Kitchin N Absalon J Gurtman A Lockhart S. Safety and efficacy of the Bnt162b2 mrna covid-19 vaccine. N Engl J Med (2020) 383(27):2603–15. doi: 10.1056/NEJMoa2034577 PMC7745181 33301246

[118] Thomas SJ Moreira ED Jr. Kitchin N Absalon J Gurtman A Lockhart S. Safety and efficacy of the Bnt162b2 mrna covid-19 vaccine through 6 months. N Engl J Med (2021) 385(19):1761–73. doi: 10.1056/NEJMoa2110345 PMC8461570 34525277

[119] Frenck RW Jr. Klein NP Kitchin N Gurtman A Absalon J Lockhart S. Safety, immunogenicity, and efficacy of the Bnt162b2 covid-19 vaccine in adolescents. N Engl J Med (2021) 385(3):239–50. doi: 10.1056/NEJMoa2107456 PMC8174030 34043894

[120] Munro APS Janani L Cornelius V Aley PK Babbage G Baxter D. Safety and immunogenicity of seven covid-19 vaccines as a third dose (Booster) following two doses of Chadox1 ncov-19 or Bnt162b2 in the uk (Cov-boost): A blinded, multicentre, randomised, controlled, phase 2 trial. Lancet (2021) 398(10318):2258–76. doi: 10.1016/s0140-6736(21)02717-3 PMC8639161 34863358

[121] Groß R Zanoni M Seidel A Conzelmann C Gilg A Krnavek D. Heterologous Chadox1 ncov-19 and Bnt162b2 prime-boost vaccination elicits potent neutralizing antibody responses and T cell reactivity against prevalent sars-Cov-2 variants. EBioMedicine (2022) 75:103761. doi: 10.1016/j.ebiom.2021.103761 34929493 PMC8682749

[122] Lopez Bernal J Andrews N Gower C Gallagher E Simmons R Thelwall S. Effectiveness of covid-19 vaccines against the B.1.617.2 (Delta) variant. N Engl J Med (2021) 385(7):585–94. doi: 10.1056/NEJMoa2108891 PMC8314739 34289274

[123] Abu-Raddad LJ Chemaitelly H Butt AA. Effectiveness of the Bnt162b2 covid-19 vaccine against the B.1.1.7 and B.1.351 variants. N Engl J Med (2021) 385(2):187–9. doi: 10.1056/NEJMc2104974 PMC8117967 33951357

[124] Bergman P Blennow O Hansson L Mielke S Nowak P Chen P. Safety and efficacy of the mrna Bnt162b2 vaccine against sars-Cov-2 in five groups of immunocompromised patients and healthy controls in a prospective open-label clinical trial. EBioMedicine (2021) 74:103705. doi: 10.1016/j.ebiom.2021.103705 34861491 PMC8629680

[125] Firinu D Perra A Campagna M Littera R Fenu G Meloni F. Evaluation of antibody response to Bnt162b2 mrna covid-19 vaccine in patients affected by immune-mediated inflammatory diseases up to 5 months after vaccination. Clin Exp Med (2022) 22(3):477–85. doi: 10.1007/s10238-021-00771-3 PMC8570239 34741188

[126] Moyon Q Sterlin D Miyara M Anna F Mathian A Lhote R. Bnt162b2 vaccine-induced humoral and cellular responses against sars-Cov-2 variants in systemic lupus erythematosus. Ann Rheum Dis (2022) 81(4):575–83. doi: 10.1136/annrheumdis-2021-221097 PMC8494536 34607791

[127] Hadjadj J Planas D Ouedrani A Buffier S Delage L Nguyen Y. Immunogenicity of Bnt162b2 vaccine against the alpha and delta variants in immunocompromised patients with systemic inflammatory diseases. Ann Rheum Dis (2022) 81(5):720–8. doi: 10.1136/annrheumdis-2021-221508 35022159

[128] Levy I Wieder-Finesod A Litchevsky V Biber A Indenbaum V Olmer L. Immunogenicity and safety of the Bnt162b2 mrna covid-19 vaccine in people living with hiv-1. Clin Microbiol Infect (2021) 27(12):1851–5. doi: 10.1016/j.cmi.2021.07.031 PMC8382485 34438069

[129] Rueda-Fernández M Melguizo-Rodríguez L Costela-Ruiz VJ González-Acedo A Ramos-Torrecillas J Illescas-Montes R. The current status of covid-19 vaccines. a scoping review. Drug Discovery Today (2022) 27(11):103336. doi: 10.1016/j.drudis.2022.08.004 35995361 PMC9389839

[130] Ragab D Salah Eldin H Taeimah M Khattab R Salem R. The covid-19 cytokine storm; what we know so far. Front Immunol (2020) 11:1446. doi: 10.3389/fimmu.2020.01446 32612617 PMC7308649

[131] Gajjela BK Zhou MM. Calming the cytokine storm of covid-19 through inhibition of Jak2/Stat3 signaling. Drug Discovery Today (2022) 27(2):390–400. doi: 10.1016/j.drudis.2021.10.016 34743903 PMC8553370

[132] Yang L Xie X Tu Z Fu J Xu D Zhou Y. The signal pathways and treatment of cytokine storm in covid-19. Signal Transduct Target Ther (2021) 6(1):255. doi: 10.1038/s41392-021-00679-0 34234112 PMC8261820

[133] Bekele Y Sui Y Berzofsky JA. Il-7 in sars-Cov-2 infection and as a potential vaccine adjuvant. Front Immunol (2021) 12:737406. doi: 10.3389/fimmu.2021.737406 34603318 PMC8484798

[134] Saraiva M O'Garra A. The regulation of il-10 production by immune cells. Nat Rev Immunol (2010) 10(3):170–81. doi: 10.1038/nri2711 20154735

[135] Hirano T Murakami M. Covid-19: A new virus, but a familiar receptor and cytokine release syndrome. Immunity (2020) 52(5):731–3. doi: 10.1016/j.immuni.2020.04.003 PMC7175868 32325025

[136] Satarker S Tom AA Shaji RA Alosious A Luvis M Nampoothiri M. Jak-stat pathway inhibition and their implications in covid-19 therapy. Postgrad Med (2021) 133(5):489–507. doi: 10.1080/00325481.2020.1855921 33245005 PMC7784782

[137] Niedźwiedzka-Rystwej P Majchrzak A Kurkowska S Małkowska P Sierawska O Hrynkiewicz R. Immune signature of covid-19: In-depth reasons and consequences of the cytokine storm. Int J Mol Sci (2022) 23(9):4545. doi: 10.3390/ijms23094545 35562935 PMC9105989

[138] Shiomi A Usui T. Pivotal roles of gm-csf in autoimmunity and inflammation. Mediators Inflammation (2015) 2015:568543. doi: 10.1155/2015/568543 PMC4370199 25838639

[139] Potter H Kendall L Boyd T Sillau S Bosco-Lauth A Markham N. Gm-csf promotes immune response and survival in a mouse model of covid-19. Res Sq (2022). doi: 10.21203/rs.3.rs-1213395/v1

[140] Zhang C Wu Z Li JW Zhao H Wang GQ. Cytokine release syndrome in severe covid-19: Interleukin-6 receptor antagonist tocilizumab may be the key to reduce mortality. Int J Antimicrob Agents (2020) 55(5):105954. doi: 10.1016/j.ijantimicag.2020.105954 32234467 PMC7118634

[141] Saha A Sharma AR Bhattacharya M Sharma G Lee SS Chakraborty C. Tocilizumab: A therapeutic option for the treatment of cytokine storm syndrome in covid-19. Arch Med Res (2020) 51(6):595–7. doi: 10.1016/j.arcmed.2020.05.009 PMC7241374 32482373

[142] Cure E Kucuk A Cure MC. Can emapalumab be life saving for refractory, recurrent, and progressive cytokine storm caused by covid-19, which is resistant to anakinra, tocilizumab, and janus kinase inhibitors. Indian J Pharmacol (2021) 53(3):226–8. doi: 10.4103/ijp.IJP_615_20 PMC8262416 34169908

[143] Patel S Saxena B Mehta P. Recent updates in the clinical trials of therapeutic monoclonal antibodies targeting cytokine storm for the management of covid-19. Heliyon (2021) 7(2):e06158. doi: 10.1016/j.heliyon.2021.e06158 33553708 PMC7846241

[144] Calabrese LH Lenfant T Calabrese C. Cytokine storm release syndrome and the prospects for immunotherapy with covid-19, part 4: The role of jak inhibition. Cleve Clin J Med (2021). doi: 10.3949/ccjm.87a.ccc060 32847819

[145] Sharma VK Prateeksha Singh SP Singh BN Rao CV Barik SK. Nanocurcumin potently inhibits sars-Cov-2 spike protein-induced cytokine storm by deactivation of Mapk/Nf-Kb signaling in epithelial cells. ACS Appl Bio Mater (2022) 5(2):483–91. doi: 10.1021/acsabm.1c00874 35112841

[146] Tirunavalli SK Gourishetti K Kotipalli RSS Kuncha M Kathirvel M Kaur R. Dehydrozingerone ameliorates lipopolysaccharide induced acute respiratory distress syndrome by inhibiting cytokine storm, oxidative stress *Via* modulating the Mapk/Nf-Kb pathway. Phytomedicine (2021) 92:153729. doi: 10.1016/j.phymed.2021.153729 34517257 PMC8390101

[147] Suryavanshi SV Zaiachuk M Pryimak N Kovalchuk I Kovalchuk O. Cannabinoids alleviate the lps-induced cytokine storm via attenuating Nlrp3 inflammasome signaling and Tyk2-mediated Stat3 signaling pathways in vitro. Cells (2022) 11(9):1391. doi: 10.3390/cells11091391 35563697 PMC9103143

[148] Manayi A Nabavi SM Khayatkashani M Habtemariam S Khayat Kashani HR. Arglabin could target inflammasome-induced Ards and cytokine storm associated with covid-19. Mol Biol Rep (2021) 48(12):8221–5. doi: 10.1007/s11033-021-06827-7 PMC8519322 34655016

[149] Sly LM Braun P Woodcock BG. Covid-19: Cytokine storm Modulation/Blockade with oral polyvalent immunoglobulins (Pvig, Kmp01d): A potential and safe therapeutic agent (Primum nil nocere). Int J Clin Pharmacol Ther (2020) 58(12):678–86. doi: 10.5414/cp203890 33141018

[150] Molinaro R Pasto A Taraballi F Giordano F Azzi JA Tasciotti E. Biomimetic nanoparticles potentiate the anti-inflammatory properties of dexamethasone and reduce the cytokine storm syndrome: An additional weapon against covid-19? Nanomaterials (Basel) (2020) 10(11):2301. doi: 10.3390/nano10112301 PMC7699958 33233748

[151] Li H You J Yang X Wei Y Zheng L Zhao Y. Glycyrrhetinic acid: A potential drug for the treatment of covid-19 cytokine storm. Phytomedicine (2022) 102:154153. doi: 10.1016/j.phymed.2022.154153 35636166 PMC9098921

[152] Yeh YC Doan LH Huang ZY Chu LW Shi TH Lee YR. Honeysuckle (Lonicera japonica) and huangqi (Astragalus membranaceus) suppress sars-Cov-2 entry and covid-19 related cytokine storm in vitro. Front Pharmacol (2021) 12:765553. doi: 10.3389/fphar.2021.765553 35401158 PMC8990830

[153] You J Li H Fan P Yang X Wei Y Zheng L. Inspiration for covid-19 treatment: Network analysis and experimental validation of baicalin for cytokine storm. Front Pharmacol (2022) 13:853496. doi: 10.3389/fphar.2022.853496 35350754 PMC8957998

[154] Nalbandian A Sehgal K Gupta A Madhavan MV McGroder C Stevens JS. Post-acute covid-19 syndrome. Nat Med (2021) 27(4):601–15. doi: 10.1038/s41591-021-01283-z PMC8893149 33753937

[155] Al-Aly Z Xie Y Bowe B. High-dimensional characterization of post-acute sequelae of covid-19. Nature (2021) 594(7862):259–64. doi: 10.1038/s41586-021-03553-9 33887749

[156] Chen C Haupert SR Zimmermann L Shi X Fritsche LG Mukherjee B. Global prevalence of post covid-19 condition or long covid: A meta-analysis and systematic review. J Infect Dis (2022). doi: 10.1093/infdis/jiac136 PMC9047189 35429399

[157] Davis HE Assaf GS McCorkell L Wei H Low RJ Re'em Y. Characterizing long covid in an international cohort: 7 months of symptoms and their impact. EClinicalMedicine (2021) 38:101019. doi: 10.1016/j.eclinm.2021.101019 34308300 PMC8280690

[158] Berger Z Altiery DEJV Assoumou SA Greenhalgh T. Long covid and health inequities: The role of primary care. Milbank Q (2021) 99(2):519–41. doi: 10.1111/1468-0009.12505 PMC8241274 33783907

[159] Callard F Perego E. How and why patients made long covid. Soc Sci Med (2021) 268:113426. doi: 10.1016/j.socscimed.2020.113426 33199035 PMC7539940

[160] Soriano JB Murthy S Marshall JC Relan P Diaz JV. A clinical case definition of post-Covid-19 condition by a Delphi consensus. Lancet Infect Dis (2022) 22(4):e102–e7. doi: 10.1016/s1473-3099(21)00703-9 PMC8691845 34951953

[161] Mandal S Barnett J Brill SE Brown JS Denneny EK Hare SS. 'Long-covid': A cross-sectional study of persisting symptoms, biomarker and imaging abnormalities following hospitalisation for covid-19. Thorax (2021) 76(4):396–8. doi: 10.1136/thoraxjnl-2020-215818 PMC7615158 33172844

[162] Dennis A Wamil M Alberts J Oben J Cuthbertson DJ Wootton D. Multiorgan impairment in low-risk individuals with post-Covid-19 syndrome: A prospective, community-based study. BMJ Open (2021) 11(3):e048391. doi: 10.1136/bmjopen-2020-048391 PMC8727683 33785495

[163] Dani M Dirksen A Taraborrelli P Torocastro M Panagopoulos D Sutton R. Autonomic dysfunction in 'Long covid': Rationale, physiology and management strategies. Clin Med (Lond) (2021) 21(1):e63–e7. doi: 10.7861/clinmed.2020-0896 PMC7850225 33243837

[164] Sun B Tang N Peluso MJ Iyer NS Torres L Donatelli JL. Characterization and biomarker analyses of post-Covid-19 complications and neurological manifestations. Cells (2021) 10(2):386. doi: 10.3390/cells10020386 33668514 PMC7918597

[165] Taquet M Geddes JR Husain M Luciano S Harrison PJ. 6-month neurological and psychiatric outcomes in 236 379 survivors of covid-19: A retrospective cohort study using electronic health records. Lancet Psychiatry (2021) 8(5):416–27. doi: 10.1016/s2215-0366(21)00084-5 PMC8023694 33836148

[166] Schuijt TJ Lankelma JM Scicluna BP de Sousa e Melo F Roelofs JJ de Boer JD. The gut microbiota plays a protective role in the host defence against pneumococcal pneumonia. Gut (2016) 65(4):575–83. doi: 10.1136/gutjnl-2015-309728 PMC4819612 26511795

[167] Zarei M Bose D Nouri-Vaskeh M Tajiknia V Zand R Ghasemi M. Long-term side effects and lingering symptoms post covid-19 recovery. Rev Med Virol (2022) 32(3):e2289. doi: 10.1002/rmv.2289 34499784 PMC8646420

[168] Mumtaz A Sheikh AAE Khan AM Khalid SN Khan J Nasrullah A. Covid-19 vaccine and long covid: A scoping review. Life (Basel) (2022) 12(7):1066. doi: 10.3390/life12071066 35888154 PMC9324565

[169] Kuodi P Gorelik Y Zayyad H Wertheim O Wiegler KB Jabal KA. Association between Bnt162b2 Vaccination and Reported Incidence of Post-Covid-19 Symptoms: Cross-Sectional Study 2020-21, Israel. npj Vaccines (2022) 7(1):101. doi: 10.1101/2022.01.05.22268800 36028498 PMC9411827

[170] Ayoubkhani D Bermingham C Pouwels KB Glickman M Nafilyan V Zaccardi F. Trajectory of long covid symptoms after covid-19 vaccination: Community based cohort study. Bmj (2022) 377:e069676. doi: 10.1136/bmj-2021-069676 35584816 PMC9115603

[171] Zisis SN Durieux JC Mouchati C Perez JA McComsey GA. The protective effect of coronavirus disease 2019 (Covid-19) vaccination on postacute sequelae of covid-19: A multicenter study from a Large national health research network. Open Forum Infect Dis (2022) 9(7):ofac228. doi: 10.1093/ofid/ofac228 35818362 PMC9129153

[172] Hung IF Lung KC Tso EY Liu R Chung TW Chu MY. Triple combination of interferon beta-1b, lopinavir-ritonavir, and ribavirin in the treatment of patients admitted to hospital with covid-19: An open-label, randomised, phase 2 trial. Lancet (2020) 395(10238):1695–704. doi: 10.1016/s0140-6736(20)31042-4 PMC7211500 32401715

[173] Dastan F Nadji SA Saffaei A Marjani M Moniri A Jamaati H. Subcutaneous administration of interferon beta-1a for covid-19: A non-controlled prospective trial. Int Immunopharmacol (2020) 85:106688. doi: 10.1016/j.intimp.2020.106688 32544867 PMC7275997

[174] Liu B Li M Zhou Z Guan X Xiang Y. Can we use interleukin-6 (Il-6) blockade for coronavirus disease 2019 (Covid-19)-Induced cytokine release syndrome (Crs)? J Autoimmun (2020) 111:102452. doi: 10.1016/j.jaut.2020.102452 32291137 PMC7151347

[175] Ullah S Abid R Haider S Khuda F Albadrani GM Abdulhakim JA. Assessment of tocilizumab (Humanized monoclonal antibody) for therapeutic efficacy and clinical safety in patients with coronavirus disease (Covid-19). Medicina (Kaunas) (2022) 58(8):1076. doi: 10.3390/medicina58081076 36013543 PMC9412443

[176] Murillo M Lomiguen C Terrell M King A Lin J Ferretti S. Effect of sars Cov2-neutralizing monoclonal antibody on hospitalization and mortality in long-term care facility residents. Aging Dis (2022) 13(5):1523–31. doi: 10.14336/ad.2022.0205 PMC9466966 36186125

[177] Ji Y Zhang Q Cheng L Ge J Wang R Fang M. Preclinical characterization of amubarvimab and romlusevimab, a pair of non-competing neutralizing monoclonal antibody cocktail, against sars-Cov-2. Front Immunol (2022) 13:980435. doi: 10.3389/fimmu.2022.980435 36189212 PMC9518701

[178] Runfeng L Yunlong H Jicheng H Weiqi P Qinhai M Yongxia S. Lianhuaqingwen exerts anti-viral and anti-inflammatory activity against novel coronavirus (Sars-Cov-2). Pharmacol Res (2020) 156:104761. doi: 10.1016/j.phrs.2020.104761 32205232 PMC7102548

[179] Xu J Liu H Fan Y Ji B. Traditional Chinese medicine is effective for covid-19: A systematic review and meta-analysis. Med Nov Technol Devices (2022) 16:100139. doi: 10.1016/j.medntd.2022.100139 35527852 PMC9066964

[180] Cai J Wang YL Sheng XD Zhang L Lv X. Shufeng jiedu capsule inhibits inflammation and apoptosis by activating A2aar and inhibiting nf-Kb to alleviate lps-induced Ali. J Ethnopharmacol (2022) 298:115661. doi: 10.1016/j.jep.2022.115661 36002086 PMC9392900

[181] Mei J Zhou Y Yang X Zhang F Liu X Yu B. Active components in ephedra sinica stapf disrupt the interaction between Ace2 and sars-Cov-2 rbd: Potent covid-19 therapeutic agents. J Ethnopharmacol (2021) 278:114303. doi: 10.1016/j.jep.2021.114303 34102269 PMC8178536

[182] Liu H Ye F Sun Q Liang H Li C Li S. Scutellaria baicalensis extract and baicalein inhibit replication of sars-Cov-2 and its 3c-like protease in vitro. J Enzyme Inhib Med Chem (2021) 36(1):497–503. doi: 10.1080/14756366.2021.1873977 33491508 PMC7850424

[183] Chien LH Deng JS Jiang WP Chen CC Chou YN Lin JG. Study on the potential of sanghuangporus sanghuang and its components as covid-19 spike protein receptor binding domain inhibitors. BioMed Pharmacother (2022) 153:113434. doi: 10.1016/j.biopha.2022.113434 36076488 PMC9288968

[184] Wang Z Wang N Yang L Song XQ. Bioactive natural products in covid-19 therapy. Front Pharmacol (2022) 13:926507. doi: 10.3389/fphar.2022.926507 36059994 PMC9438897

[185] Schoenmaker L Witzigmann D Kulkarni JA Verbeke R Kersten G Jiskoot W. Mrna-lipid nanoparticle covid-19 vaccines: Structure and stability. Int J Pharm (2021) 601:120586. doi: 10.1016/j.ijpharm.2021.120586 33839230 PMC8032477

[186] Ullah A Qazi J Rahman L Kanaras AG Khan WS Hussain I. Nanoparticles-assisted delivery of antiviral-sirna as inhalable treatment for human respiratory viruses: A candidate approach against sars-Cov-2. Nano Sel (2020) 1(6):612–21. doi: 10.1002/nano.202000125 PMC7675679 34485978

[187] Zachar O. Nanomedicine formulations for respiratory infections by inhalation delivery: Covid-19 and beyond. Med Hypotheses (2022) 159:110753. doi: 10.1016/j.mehy.2021.110753 35002023 PMC8721932

[188] Gunathilake T Ching YC Uyama H Hai ND Chuah CH. Enhanced curcumin loaded nanocellulose: A possible inhalable nanotherapeutic to treat covid-19. Cellulose (Lond) (2022) 29(3):1821–40. doi: 10.1007/s10570-021-04391-8 PMC8725427 35002106

[189] Tulbah AS Lee WH. Physicochemical characteristics and in vitro Toxicity/Anti-Sars-Cov-2 activity of favipiravir solid lipid nanoparticles (Slns). Pharm (Basel) (2021) 14(10):1059. doi: 10.3390/ph14101059 PMC8540419 34681283

[190] Jin H Zhao Z Lan Q Zhou H Mai Z Wang Y. Nasal delivery of Hesperidin/Chitosan nanoparticles suppresses cytokine storm syndrome in a mouse model of acute lung injury. Front Pharmacol (2020) 11:592238. doi: 10.3389/fphar.2020.592238 33584267 PMC7873598

[191] Rabiei M Kashanian S Samavati SS Derakhshankhah H Jamasb S McInnes SJP. Characteristics of sars-Cov2 that may be useful for nanoparticle pulmonary drug delivery. J Drug Target (2022) 30(3):233–43. doi: 10.1080/1061186x.2021.1971236 34415800

[192] Mohamed Y El-Maradny YA Saleh AK Nayl AA El-Gendi H El-Fakharany EM. A comprehensive insight into current control of covid-19: Immunogenicity, vaccination, and treatment. BioMed Pharmacother (2022) 153:113499. doi: 10.1016/j.biopha.2022.113499 36076589 PMC9343749

[193] Choudhary OP Priyanka Ahmed JQ Mohammed TA Singh I Rodriguez-Morales AJ. Heterologous prime-boost vaccination against covid-19: Is it safe and reliable? Hum Vaccin Immunother (2021) 17(12):5135–8. doi: 10.1080/21645515.2021.2007015 PMC8726007 34898381

[194] Labouta HI Langer R Cullis PR Merkel OM Prausnitz MR Gomaa Y. Role of drug delivery technologies in the success of covid-19 vaccines: A perspective. Drug Delivery Transl Res (2022) 12(11):2581–8. doi: 10.1007/s13346-022-01146-1 PMC8923087 35290656

[195] Zhang Q Meng Y Wang K Zhang X Chen W Sheng J. Inflammation and antiviral immune response associated with severe progression of covid-19. Front Immunol (2021) 12:631226. doi: 10.3389/fimmu.2021.631226 33679778 PMC7930228

[196] Tahmasebi S Saeed BQ Temirgalieva E Yumashev AV El-Esawi MA Navashenaq JG. Nanocurcumin improves treg cell responses in patients with mild and severe sars-Cov2. Life Sci (2021) 276:119437. doi: 10.1016/j.lfs.2021.119437 33789145 PMC8005319

[197] Rarani FZ Rashidi B Jafari Najaf Abadi MH Hamblin MR Reza Hashemian SM Mirzaei H. Cytokines and micrornas in sars-Cov-2: What do we know? Mol Ther Nucleic Acids (2022) 29:219–42. doi: 10.1016/j.omtn.2022.06.017 PMC9233348 35782361

