# Supplementary material for: Uncovering the information immunology journals transmitted for COVID-19: A bibliometric and visualization analysis
Source: Front Immunol. 2022 Oct 31;13:1035151. doi: 10.3389/fimmu.2022.1035151 (PMC9670819; doi:10.3389/fimmu.2022.1035151)
Supplement: Supplementary Table 1 — The top 25 institutions with the most publications. [file Table_1.docx]

|  | Part A | | | | Part B | | | |
| --- | --- | --- | --- | --- | --- | --- | --- | --- |
| Rank | Institutions | Country | No. of Publications | Centrality | Institutions | Country | No. of Publications | Centrality |
| 1 | Univ Hong Kong | CHINA | 75 | 0.04 | Ctr Dis Control & Prevent | USA | 131 | 0.05 |
| 2 | Ctr Dis Control & Prevent | USA | 63 | 0.06 | Harvard Med Sch | USA | 109 | 0.08 |
| 3 | Harvard Med Sch | USA | 63 | 0.12 | Univ Hong Kong | CHINA | 74 | 0.07 |
| 4 | Emory Univ | USA | 46 | 0.07 | Emory Univ | USA | 74 | 0.05 |
| 5 | Chinese Acad Sci | CHINA | 42 | 0.07 | Johns Hopkins Univ | USA | 68 | 0.06 |
| 6 | Univ Washington | USA | 42 | 0.08 | Huazhong Univ Sci & Technol | CHINA | 66 | 0.05 |
| 7 | NIAID | USA | 40 | 0.13 | Univ Washington | USA | 65 | 0.06 |
| 8 | Univ Calif San Francisco | USA | 36 | 0.05 | Univ Oxford | UK | 62 | 0.05 |
| 9 | Stanford Univ | USA | 35 | 0.00 | Univ Calif San Francisco | USA | 61 | 0.03 |
| 10 | Univ Oxford | ENGLAND | 34 | 0.06 | Univ Milan | ITALY | 58 | 0.01 |
| 11 | Johns Hopkins Univ | USA | 34 | 0.07 | Imperial Coll London | UK | 57 | 0.02 |
| 12 | Huazhong Univ Sci & Technol | CHINA | 33 | 0.08 | Tel Aviv Univ | ISRAEL | 48 | 0.02 |
| 13 | Univ Penn | USA | 33 | 0.11 | NIAID | USA | 46 | 0.05 |
| 14 | Univ Calif San Diego | USA | 31 | 0.12 | Columbia Univ | USA | 46 | 0.02 |
| 15 | Fudan Univ | CHINA | 31 | 0.02 | Univ Michigan | USA | 45 | 0.09 |
| 16 | Imperial Coll London | ENGLAND | 31 | 0.02 | Johns Hopkins Bloomberg Sch Publ Hlth | USA | 45 | 0.02 |
| 17 | UCL | ENGLAND | 30 | 0.3 | Stanford Univ | USA | 44 | 0.05 |
| 18 | Johns Hopkins Bloomberg Sch Publ Hlth | USA | 30 | 0.02 | Icahn Sch Med Mt Sinai | USA | 44 | 0.05 |
| 19 | Icahn Sch Med Mt Sinai | USA | 30 | 0.02 | Minist Hlth | NA | 43 | 0.05 |
| 20 | Queen Mary Hosp | ENGLAND | 29 | 0.00 | London Sch Hyg & Trop Med | UK | 42 | 0.04 |
| 21 | Brigham & Womens Hosp | USA | 26 | 0.00 | Natl & Kapodistrian Univ Athens | GREECE | 42 | 0.00 |
| 22 | Washington Univ | USA | 25 | 0.02 | Fudan Univ | CHINA | 42 | 0.01 |
| 23 | Chinese Acad Med Sci & Peking Union Med Coll | CHINA | 25 | 0.00 | Peking Univ | CHINA | 42 | 0.02 |
| 24 | Harvard TH Chan Sch Publ Hlth | USA | 24 | 0.00 | Univ Pittsburgh | USA | 42 | 0.03 |
| 25 | Wuhan Univ | CHINA | 24 | 0.05 | Univ Melbourne | ALSTRALIA | 39 | 0.01 |

Table S1 The top 25 institutions with the most publications

Abbreviations: UCL, University College London; NIAID, National Institute of Allergy and Infectious Diseases
