# Supplementary material for: Uncovering the information immunology journals transmitted for COVID-19: A bibliometric and visualization analysis
Source: Front Immunol. 2022 Oct 31;13:1035151. doi: 10.3389/fimmu.2022.1035151 (PMC9670819; doi:10.3389/fimmu.2022.1035151)
Supplement: Supplementary Table 2 — Top 10 highly co-cited references. [file Table_2.docx]

Table S2 Top 10 highly co-cited references

|  |  | First Authors | Title | Journals | Citations |
| --- | --- | --- | --- | --- | --- |
| Part A | 1 | Huang CL | Clinical features of patients infected with 2019 novel coronavirus in Wuhan, China | LANCET | 137 |
|  | 2 | Zhu N | A Novel Coronavirus from Patients with Pneumonia in China, 2019 | NEW ENGL J MED | 120 |
|  | 3 | Zhou F | Clinical course and risk factors for mortality of adult inpatients with COVID-19 in Wuhan, China: a retrospective cohort study | LANCET | 108 |
|  | 4 | Polack FP | Safety and Efficacy of the BNT162b2 mRNA Covid-19 Vaccine | NEW ENGL J MED | 108 |
|  | 5 | Guan W | Clinical Characteristics of Coronavirus Disease 2019 in China | NEW ENGL J MED | 100 |
|  | 6 | Grifoni A | Targets of T Cell Responses to SARS-CoV-2 Coronavirus in Humans with COVID-19 Disease and Unexposed Individuals | CELL | 94 |
|  | 7 | Baden LR | Efficacy and Safety of the mRNA-1273 SARS-CoV-2 Vaccine | NEW ENGL J MED | 92 |
|  | 8 | Hoffmann M | SARS-CoV-2 Cell Entry Depends on ACE2 and TMPRSS2 and Is Blocked by a Clinically Proven Protease Inhibitor | CELL | 91 |
|  | 9 | Zhou P | Addendum: A pneumonia outbreak associated with a new coronavirus of probable bat origin | NATURE | 87 |
|  | 10 | Chan JFW | A familial cluster of pneumonia associated with the 2019 novel coronavirus indicating person-to-person transmission: a study of a family cluster | LANCET | 72 |
| Part B | 1 | Polack FP | Safety and Efficacy of the BNT162b2 mRNA Covid-19 Vaccine | NEW ENGL J MED | 628 |
|  | 2 | Huang CL | Clinical features of patients infected with 2019 novel coronavirus in Wuhan, China | LANCET | 461 |
|  | 3 | Baden LR | Efficacy and Safety of the mRNA-1273 SARS-CoV-2 Vaccine | NEW ENGL J MED | 401 |
|  | 4 | Zhu N | A Novel Coronavirus from Patients with Pneumonia in China, 2019 | NEW ENGL J MED | 349 |
|  | 5 | Hoffmann M | SARS-CoV-2 Cell Entry Depends on ACE2 and TMPRSS2 and Is Blocked by a Clinically Proven Protease Inhibitor | CELL | 312 |
|  | 6 | Zhou F | Clinical course and risk factors for mortality of adult inpatients with COVID-19 in Wuhan, China: a retrospective cohort study | LANCET | 309 |
|  | 7 | Guan W | Clinical Characteristics of Coronavirus Disease 2019 in China | NEW ENGL J MED | 273 |
|  | 8 | Zhao JJ | Antibody Responses to SARS-CoV-2 in Patients With Novel Coronavirus Disease 2019 | CLIN INFECT DIS | 231 |
|  | 9 | Grifoni A | Targets of T Cell Responses to SARS-CoV-2 Coronavirus in Humans with COVID-19 Disease and Unexposed Individuals | CELL | 224 |
|  | 10 | Lazarus JV | A global survey of potential acceptance of a COVID-19 vaccine | NAT MED | 216 |
